# External Quality Assessment of SARS-CoV-2 Sequencing: an ESGMD-SSM Pilot Trial across 15 European Laboratories

**DOI:** 10.1128/JCM.01698-21

**Published:** 2022-01-19

**Authors:** Fanny Wegner, Tim Roloff, Michael Huber, Samuel Cordey, Alban Ramette, Yannick Gerth, Claire Bertelli, Madlen Stange, Helena M. B. Seth-Smith, Alfredo Mari, Karoline Leuzinger, Lorenzo Cerutti, Keith Harshman, Ioannis Xenarios, Philippe Le Mercier, Pascal Bittel, Stefan Neuenschwander, Onya Opota, Jonas Fuchs, Marcus Panning, Charlotte Michel, Marie Hallin, Thomas Demuyser, Ricardo De Mendonca, Paul Savelkoul, Jozef Dingemans, Brian van der Veer, Stefan A. Boers, Eric C. J. Claas, Jordy P. M. Coolen, Willem J. G. Melchers, Marianne Gunell, Teemu Kallonen, Tytti Vuorinen, Antti J. Hakanen, Eva Bernhoff, Marit Andrea Klokkhammer Hetland, Hadar Golan Berman, Sheera Adar, Jacob Moran-Gilad, Dana G. Wolf, Stephen L. Leib, Oliver Nolte, Laurent Kaiser, Stefan Schmutz, Verena Kufner, Maryam Zaheri, Alexandra Trkola, Hege Vangstein Aamot, Hans H. Hirsch, Gilbert Greub, Adrian Egli

**Affiliations:** a Applied Microbiology Research, Department of Biomedicine, University of Basel, Basel, Switzerland; b Clinical Bacteriology and Mycology, University Hospital Basel, Basel, Switzerland; c Swiss Institute of Bioinformatics, Basel, Switzerland; d Institute of Medical Virology, University of Zurich, Zurich, Switzerland; e Laboratory of Virology, University Hospital Geneva, Geneva, Switzerland; f Institute for Infectious Diseases, University of Bern, Bern, Switzerland; g Center for Laboratory Medicine, Saint Gall, Switzerland; h Institute of Microbiology, Lausanne University Hospital, Lausanne, Switzerland; i Institute of Microbiology, University of Lausanne, Lausanne, Switzerland; j Clinical Virology, University Hospital Basel, Basel, Switzerland; k Transplantation and Clinical Virology, Department of Biomedicine, University of Basel, Basel, Switzerland; l Health 2030 Genome Center, Geneva, Switzerland; m SwissProt Group, Swiss Institute of Bioinformatics, Geneva, Switzerland; n Institute of Virology, Medical Center-University of Freiburg, Faculty of Medicine, University of Freiburg, Freiburg, Germany; o Department of Microbiology, Laboratoire Hospitalier Universitaire de Bruxelles, Brussels, Belgium; p Department of Microbiology and Infection Control, Universitair Ziekenhuis Brussel, Vrije Universiteit Brussel, Brussels, Belgium; q Université Libre de Bruxelles (ULB), Brussels, Belgium; r Department of Medical Microbiology, Maastricht University, Maastricht, Netherlands; s Department of Medical Microbiology, Leiden University Medical Center, Leiden, Netherlands; t Department of Medical Microbiology, Radboud University Medical Center, Nijmegen, Netherlands; u Department of Clinical Microbiology, Turku University Hospital, Turku, Finland; v Department of Clinical Microbiology, University of Turku, Turku, Finland; w Department of Medical Microbiology, Stavanger University Hospital, Stavanger, Norway; x Clinical Virology Unit, Department of Clinical Microbiology and Infectious Diseases, Hadassah University Hospital, Jerusalem, Israel; y Lautenberg Center for General and Tumor Immunology, The Hebrew University Faculty of Medicine, Jerusalem, Israel; z Department of Microbiology and Molecular Genetics, Institute for Medical Research Israel Canada, The Hebrew University Faculty of Medicine, Jerusalem, Israel; aa School of Public Health, Faculty of Health Sciences, Ben-Gurion University of the Negev, Beer Sheva, Israel; bb Department of Microbiology and Infection Control, Akershus University Hospital, Lørenskog, Norway; cc Department of Clinical Molecular Biology (EPIGEN), Akershus University Hospital and University of Oslo, Lørenskog, Norway; dd Infectious Diseases and Hospital Epidemiology, University Hospital Basel, Basel, Switzerland; ee Infectious Diseases and Hospital Epidemiology, University of Basel, Basel, Switzerland; ff ESCMID Study Group for Genomic and Molecular Diagnostics (ESGMD), Basel, Switzerland; Cepheid

**Keywords:** NGS, external quality assessment, ring trial, whole-genome sequencing

## Abstract

This first pilot trial on external quality assessment (EQA) of severe acute respiratory syndrome coronavirus 2 (SARS-CoV-2) whole-genome sequencing, initiated by the European Society of Clinical Microbiology and Infectious Diseases (ESCMID) Study Group for Genomic and Molecular Diagnostics (ESGMD) and the Swiss Society for Microbiology (SSM), aims to build a framework between laboratories in order to improve pathogen surveillance sequencing. Ten samples with various viral loads were sent out to 15 clinical laboratories that had free choice of sequencing methods and bioinformatic analyses. The key aspects on which the individual centers were compared were the identification of (i) single nucleotide polymorphisms (SNPs) and indels, (ii) Pango lineages, and (iii) clusters between samples. The participating laboratories used a wide array of methods and analysis pipelines. Most were able to generate whole genomes for all samples. Genomes were sequenced to various depths (up to a 100-fold difference across centers). There was a very good consensus regarding the majority of reporting criteria, but there were a few discrepancies in lineage and cluster assignments. Additionally, there were inconsistencies in variant calling. The main reasons for discrepancies were missing data, bioinformatic choices, and interpretation of data. The pilot EQA was overall a success. It was able to show the high quality of participating laboratories and provide valuable feedback in cases where problems occurred, thereby improving the sequencing setup of laboratories. A larger follow-up EQA should, however, improve on defining the variables and format of the report. Additionally, contamination and/or minority variants should be a further aspect of assessment.

## INTRODUCTION

Whole-genome sequencing (WGS) of severe acute respiratory syndrome coronavirus 2 (SARS-CoV-2) isolates has been used in many countries mainly to determine (i) specific viral lineages and (ii) the molecular epidemiological context. WGS will become increasingly important both as a typing technology in virological routine diagnostics of individual patients and for epidemiological surveillance. The European Centre for Disease Prevention and Control (ECDC) recently published a document to support the usage and implementation of WGS of SARS-CoV-2 in European countries (1).

Quality management is a central element for ensuring accurate and robust laboratory results for both routine diagnostic and reference laboratories. Internal and external controls are integral to the assessment of quality, e.g., in an ISO-accredited environment. In particular, external quality assessments (EQAs) represent a cornerstone in introducing new test methods, capacity building, and ensuring a baseline quality level. This is even more important in a pandemic situation, where a novel, previously unknown pathogen necessitates prompt development, validation, and rollout of assays for which microbiological expertise and diagnostic knowledge are limited. In this context, EQAs can ensure and improve testing quality and result comparability. They also allow, if sufficiently scaled, the comparison of the test performances of in-house-developed and commercial assays.

To date, no EQA results have been reported focusing on WGS of SARS-CoV-2, although some publications have shared quality aspects of a single center’s experiences (2, 3). Along these lines, individual centers in Switzerland have reported protocols on WGS with different epidemiological questions (4, 5). In the past, the Swiss Institute of Bioinformatics has coordinated EQAs for viral metagenomics (6) and bacterial typing (7), which is an important first step in the capacity forming of WGS technology between diagnostic laboratories. Many other European countries are following suit.

For this reason, the European Society of Clinical Microbiology and Infectious Diseases (ESCMID) Study Group for Genomic and Molecular Diagnostics (ESGMD) and the Swiss Society of Microbiology (SSM) aimed to conduct a first EQA pilot trial focusing on SARS-CoV-2 WGS with a focus on three key aspects of genome analysis: (i) identification of single nucleotide polymorphisms (SNPs) and deletions, (ii) identification of Pango lineages (8), and (iii) assessing genomic relatedness using a molecular epidemiological approach.

The aim is to exchange knowledge and build a framework between diagnostic laboratories in order to improve quality for the continuing demands for high-quality genomes to address epidemiological questions during an ongoing pandemic.

## MATERIALS AND METHODS

### Design of the external quality assessment.

The EQA was designed such that each laboratory could choose its own sequencing method as well as bioinformatic analysis. This introduces variability and makes disentangling methodological effects more difficult but best reflects clinical reality. Moreover, it provides direct feedback to laboratories concerning their sequencing pipeline.

An overview of the individual analysis pipelines is shown in Table 1, and a full description can be found in the supplemental material.

**TABLE 1:** Summary of the methods used by the participating centers^*a*^

Center	Primer panel (manufacturer)	Sequencing technology	Bioinformatics pipeline	Reference(s)
1	Artic nCoV-2019 v3	Illumina MiSeq, 150-bp SE	SmaltAlign	14
2	Artic nCoV-2019 v3	Nanopore	Artic bioinformatics pipeline v1.1.3	15
3	Artic nCoV-2019 v3	Illumina MiSeq, 150-bp PE	virSEAK pipeline (JSI Medical Systems)	
4	CleanPlex SARS-CoV-2 (Paragon Genomics)	Illumina MiSeq, 150-bp PE	GENCOV	29
5	Artic nCoV-2019 v3	Illumina MiSeq, 150-bp PE	Custom Galaxy pipeline	16, 17
6	Custom	Nanopore	MACOVID pipeline	18, 19
7	EasySeq RC-PCR SARS-CoV-2 (NimaGen)	Illumina, MiniSeq, 150-bp PE	Custom pipeline	20
8	EasySeq RC-PCR SARS-CoV-2 (NimaGen)	Illumina, MiniSeq, 150-bp PE	EasySeq pipeline	21, 22
9	Midnight primer panel (IDT)	Nanopore	Artic bioinformatics pipeline	15
10	Artic nCoV-2019 v3	Nanopore	Artic bioinformatics pipeline	15, 20
11	Artic nCoV-2019 v3	Nanopore	SusCovONT	23
12	QIAseq SARS-CoV-2 primer panel (Qiagen)	Illumina MiniSeq, 150-bp PE	Illumina BaseSpace Dragen COVID lineage	
13	Illumina COVIDSeq test	Illumina NovaSeq, 50-bp PE	Health 2030 Genome Center in Geneva pipeline	24
14	Illumina COVIDSeq test	Illumina NovaSeq, 150-bp PE	Custom pipeline	25, 26
15	Artic nCoV-2019 v3	Illumina NextSeq, 150-bp PE	COVGAP	4, 27, 28

a A detailed method description by each center can be found in the supplemental material. SE, single end; PE, paired end; RC-PCR, reverse complement PCR.

The desired key aspects for the EQA (SNPs/indels, Pango lineage assignment, and cluster assignment) as well as additional features such as read depth and percentage of missing data were reported back to the sequencing team at the University Hospital Basel (coordinating center for this pilot study).

### Samples.

Large quantities of virus suspensions were needed for the EQA. For this reason, it was decided to culture the virus to generate enough material. Vero76 cells were grown in Dulbecco’s modified Eagle’s medium (DMEM) (10% fetal bovine serum, 1% glutamine) in flat-bottom 96-well plates (Thermo Fisher Scientific, MA, USA). One hundred microliters of SARS-CoV-2-positive naso-oropharyngeal fluids was added, and cells were incubated for 48 h at 37°C. The cell culture supernatants were harvested, and SARS-CoV-2 RNA was quantified using the laboratory-developed Basel-SCoV2-112bp nucleic acid test (NAT), as described previously (9), targeting specific viral sequences of the spike glycoprotein S gene.

A total of 10 samples (named NGS1 to -10) of the cell culture supernatants were frozen and shipped on dry ice to participating laboratories. The viral isolates originated from routine diagnostic samples from Clinical Virology, University Hospital Basel, reflecting diverse epidemiological backgrounds. The cell culture supernatants used contained a range of viral loads of SARS-CoV-2, reflecting viral loads typically observed in routine diagnostics of acutely ill coronavirus disease 2019 (COVID-19) patients (see Table S1 in the supplemental material). To ensure that no changes occurred during culture, both primary material and the cell culture supernatant were sequenced and compared; the resulting sequences were identical (results not shown).

### Assessment of variant calling.

SNPs, compared to the reference Wuhan-Hu-1 strain, were assessed as reported (usually in the form of a list of variants). In order to compare results across centers and samples, a score was developed. As there is no “correct solution” to compare results against, a majority consensus approach was chosen; i.e., an SNP/indel was considered correct if the majority of laboratories detected it (ignoring missing data). If the correct base was called, a score of 1 was given per site. Incorrect base calls were scored as −1; missing data received a score of 0. If an ambiguous base was called where a true SNP occurred and the correct base was included in the ambiguity code (IUPAC), a score of 0.5 was given. Otherwise, reported ambiguous sites were not counted as SNPs. In the case of deletions that were present but not reported, we chose to set the score to −1 given that centers were instructed to report deletions and that a failure to report could be an artifact of the bioinformatics pipeline. The score was finally normalized per sample by the number of correct SNPs.

### Assessment of lineage and cluster assignment.

The “correct answer” was again assumed to be the majority consensus. Clusters were relabeled to unify the nomenclature and compare laboratories. We did not provide a strict definition of a cluster but allowed laboratories to determine clusters based on internal criteria. In addition, no classical epidemiological metadata were provided, to help with potential interpretations.

## RESULTS

### Genome depth, coverage, and assembly.

**FIG 1:**
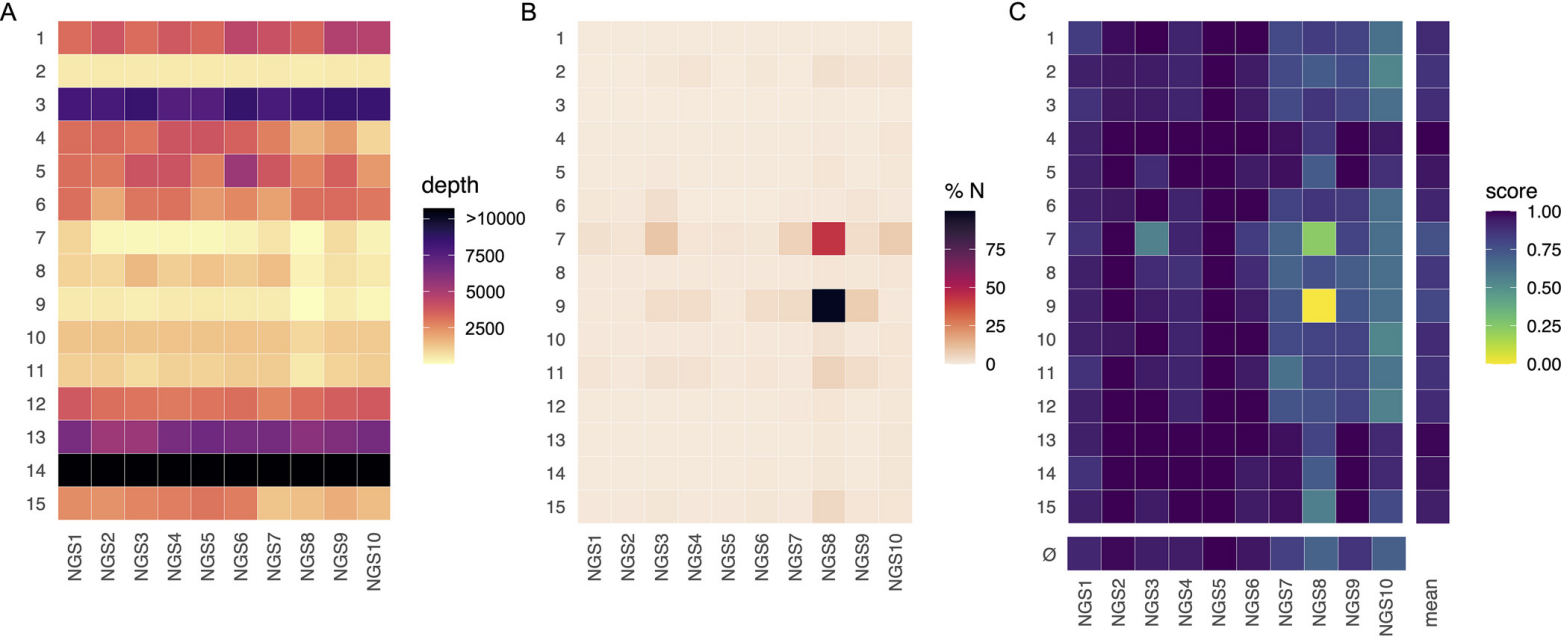
(A) Mean read depth per sample (*x* axis) and center (*y* axis). Colors have been scaled for high resolution for values of between 0 and 10,000; values larger than this are displayed in the same color. (B) Percentage of N’s in the genome per sample (*x* axis) and center (*y* axis). (C) Score for variant detection per sample (*x* axis) and center (*y* axis) as well as mean score for each center across all samples and mean score for each sample across centers (ø). The numerical values underlying each plot can be found in Tables S2 to S4 in the supplemental material.

The mean read depth per center ranged from 313× to 37,172×, which reflects a >100-fold difference across centers. However, this was mostly driven by center 14, which sequenced to an extremely high read depth (Fig. 1A; see also Table S2 in the supplemental material). Centers 7 and 9 are on the lower end of the spectrum (mean depths ± standard deviations [SD] of 325× ± 275× and 313× ± 132×, respectively), whereas all other laboratories usually sequenced to a mean depth of between 1,000× and 8,000×.

The majority of samples could be assembled to a consensus genome by all centers, with the exception of NGS8, for which assembly failed partially for center 7 and completely for center 9 as seen by the percentage of missing data shown in Fig. 1B (numeric values are shown in Table S3).

### SNPs and indels.

Variants have been assessed as reported and are displayed in Fig. S1A to J as a dot plot indicating the presence and absence of the variant. Some centers reported mixed sites using ambiguous codes, while others did not. Moreover, not all centers reported deletions. Whether these had been correctly called in the consensus genome was therefore checked for each variation and, if present, specifically marked in Fig. S1. Additionally, Table S5 lists the number of correct, wrong, and missing SNP calls for each sample and laboratory.

A variant calling score was developed in order to quantify and compare the variant calls per sample and laboratory (see Materials and Methods). The results are shown in Fig. 1C (numerical values are shown in Table S4), with average scores per sample across all centers (row marked with ø) also shown as a measure of congruence across laboratories. As expected, samples with a higher proportion of missing data produced a lower score if the affected regions harbored many variations (e.g., NGS3 by center 7, which had a coverage of 91%). Samples NGS7, -9, and -10 had many deletions, and laboratories not reporting these deletions received a correspondingly lower score. NGS8, however, was a sample with which many centers had problems. Many laboratories reported missing data for variant loci. Additionally, incorrect base calls were made, in particular by center 15 (Fig. S1H). A combination of several of these factors can in turn result in a lower mean score for a center (e.g., center 7, with an average score of 0.75) (Table S4).

### Lineage assignment.

Correct lineage assessment is of course dependent on correct SNP calling and sufficient coverage across the genome. The majority of centers assigned all samples to the correct lineage (Table 2). Two centers with the lowest mean depths failed in correctly assigning the lineage of one sample, NGS8 (B.1.177) (Table S2). Center 7, which provided a 57% complete genome (mean read depth of 39×), could assign the sample to lineage B. Rather surprisingly, the laboratory with by far the highest depth, center 14, assigned the lineages of two samples incorrectly: NGS7 and -9 were both assigned only as lineage A, as opposed to the more accurate correct solution of A.27. This was due to an outdated version of Pangolin.

**TABLE 2:** Pango lineage assignments^*a*^

Center	Lineage assignment									
	NGS1	NGS2	NGS3	NGS4	NGS5	NGS6	NGS7	NGS8	NGS9	NGS10
1	B.1.416.1	B.1.36.17	B.1.177	B.1.258	B.1.36.17	B.1.177	A.27	B.1.177	A.27	B.1.1.7
2	B.1.416.1	B.1.36.17	B.1.177	B.1.258	B.1.36.17	B.1.177	A.27	B.1.177	A.27	B.1.1.7
3	B.1.416.1	B.1.36.17	B.1.177	B.1.258	B.1.36.17	B.1.177	A.27	B.1.177	A.27	B.1.1.7
4	B.1.416.1	B.1.36.17	B.1.177	B.1.258	B.1.36.17	B.1.177	A.27	B.1.177	A.27	B.1.1.7
5	B.1.416.1	B.1.36.17	B.1.177	B.1.258	B.1.36.17	B.1.177	A.27	B.1.177	A.27	B.1.1.7
6	B.1.416.1	B.1.36.17	B.1.177	B.1.258	B1.36.17	B.1.177	A.27	B.1.177	A.27	B.1.1.7
7	B.1.416.1	B.1.36.17	B.1.177	B.1.258	B.1.36.17	B.1.177	A.27	B	A.27	B.1.1.7
8	B.1.416.1	B.1.36.17	B.1.177	B.1.258	B.1.36.17	B.1.177	A.27	B.1.177	A.27	B.1.1.7
9	B.1.416.1	B.1.36.17	B.1.177	B.1.258	B.1.36.17	B.1.177	A.27	NA	A.27	B.1.1.7
10	B.1.416.1	B.1.36.17	B.1.177	B.1.258	B.1.36.17	B.1.177	A.27	B.1.177	A.27	B.1.1.7
11	B.1.416.1	B.1.36.17	B.1.177	B.1.258	B.1.36.17	B.1.177	A.27	B.1.177	A.27	B.1.1.7
12	B.1.416.1	B.1.36.17	B.1.177	B.1.258	B.1.36.17	B.1.177	A.27	B.1.177	A.27	B.1.1.7
13	B.1.416.1	B.1.36.17	B.1.177	B.1.258	B.1.36.17	B.1.177	A.27	B.1.177	A.27	B.1.1.7
14	B.1.416.1	B.1.36.17	B.1.177	B.1.258	B.1.36.17	B.1.177	A	B.1.177	A	B.1.1.7
15	B.1.416.1	B.1.36.17	B.1.177	B.1.258	B.1.36.17	B.1.177	A.27	B.1.177	A.27	B.1.1.7

a NA, not applicable; lineage assignment was impossible. Shading highlights cases discussed in more detail in the text.

### Cluster identification.

Almost all centers reported the same clusters (Table 3). Samples NGS2 and NGS5 formed one cluster (cluster B); NGS3, NGS6, and NGS8 formed the second cluster (cluster C); and NGS7 and NGS9 formed the third cluster (cluster E).

**TABLE 3:** Cluster assignments^*a*^

Center	Cluster assignment									
	NGS1	NGS2	NGS3	NGS4	NGS5	NGS6	NGS7	NGS8	NGS9	NGS10
1	A	B	C	D	B	C	E	C	E	F
2	A	B	C	D	B	C	E	C	E	F
3	A	B	C	D	B	C	E	C	E	F
4	A	B	C	D	B	C	E	C	E	F
5	A	B	C	D	B	C	E	C	E	F
6	A	B	C	D	B	C	E	C	E	F
7	A	B	C	D	B	C	E	C*	E	F
8	A	B	C	D	B	C	E	C	E	F
9	A	B	C	D	B	C	E	NA	E	F
10	A	B	C	D	B	C	E	C	E	F
11	A	B	C	D	B	C	E	C	E	F
12	B	B	C	B	B	C	E	C	E	F
13	A	B	C	D	B	C	E	C	E	F
14	A	B	C	A	B	C	E	C	E	F
15	A	B	C	D	B	C	E	C	E	F

a NA, not applicable; cluster assignment was impossible. Shading highlights discrepant cases discussed in more detail in the text. * marks that the center reported an assumed cluster assignment based on a partial genome.

The low coverage for sample NGS8 was a challenge for the two above-mentioned centers 7 and 9. However, center 7 reported a presumed allocation into the correct cluster using the partial genome (asterisk in Table 3). Center 9 could not identify the cluster due to unsuccessful sequencing (9× mean depth [Table S2, highlighted in red]). This resulted in a too-small cluster.

Center 12 had difficulties with two samples (NGS1 and -4) and allocated them incorrectly to cluster B (together with NGS2 and -5) (Table 2, shading). This was despite them falling into different Pango lineages (Table 2). Center 14 incorrectly assigned NGS1 and NGS4 to a separate cluster (Table 2, shading), again despite differing Pango lineage assignments. However, the other clusters were correctly assigned by both laboratories.

## DISCUSSION

### Impact of methodological choices.

Given that laboratories had free choice over their experimental as well as analytical protocols, disentangling the individual effects of these differences is impossible. A factor known to influence sequencing success is viral load. For example, NGS8, while having a viral load comparable to those of NGS9 and -10 (threshold cycle [*C_T_*] values of 28.4 and 28.1, respectively), was on the lower end of the spectrum (*C_T_* value of 28) (see Table S1 in the supplemental material). This could be why many centers had problems with this sample.

When grouping the sequencing methods roughly into Illumina single-end versus Illumina paired-end versus Oxford Nanopore Technologies (ONT) methods, a platform-related effect does not seem to have occurred (Fig. S2). In fact, centers 7 and 8 had very similar sequencing setups, with the exception of their analysis pipelines (Table 1). Center 8, however, was able to sequence to a greater depth and was therefore better able to perform accurate genomic analyses as it achieved overall higher coverage across the genome. Moreover, the small genome of SARS-CoV-2 and the lack of long repeat regions allow the use of short reads or single-end sequencing, which would be more problematic for WGS of other pathogens.

The mean depth had an effect only insofar as a too-low depth leads to too much missing data. Once a sufficient read depth had been achieved, there was no further clear correlation between the score of variant calling and depth (Fig. S3). In general, depth across the genome can be very uneven, and average depth as a measure does not fully take this into account. Technically, read depths of between 100× and 200× can be enough for genotyping. For example, samples NGS2 and -5 for center 7 have 191× and 131× coverages, respectively, as well as a small amount of missing data and a high variant calling score (Fig. 1). However, when coverage is uneven, missing data can still be an issue, even at a higher average depth (e.g., NGS10 for center 7 at 246×) (Fig. 1; Table S2). For accurately genotyping SARS-CoV-2, it is necessary to capture the entirety of the genome and not just some areas (even of biologically important areas such as the S gene) as the software used to determine the lineage built its models based on whole-genome diversity (the pangoLEARN algorithm within Pangolin) (8). It is therefore important to strive for the best coverage across the genome (i.e., a small amount of missing data), and “sufficient read depth,” as mentioned above, is therefore a function of this. More even coverage in amplicon-based sequencing can, for example, be achieved by balancing primer sets.

Instead of average depth, other factors such as variant reporting capacity, mapping quality, as well as interpretation of data play a larger role. This is an important point for diagnostic laboratories with respect to operational costs. The importance of this was highlighted by center 14, which sequenced to by far the highest depth but had difficulties with lineage and cluster assignments despite very good variant calling. Upon receiving a preliminary report, center 14 reexamined its analysis pipeline and found that it had used an outdated Pangolin and pangoLEARN version. The Pango lineage nomenclature is dynamic, meaning that the nomenclature system develops as SARS-CoV-2 evolves, and lineage definitions and names can change over time (8). The pilot EQA provided valuable feedback for the center to improve its workflows.

Cluster assignment, on the other hand, highlighted another challenge for the development of any EQA: communication and interpretation. The majority of other centers determined a cluster as a putative transmission cluster that differs between 0 and maximally 2 SNPs (thresholds vary slightly) (see supplemental methods in the supplemental material). Two centers had difficulties, which could be resolved upon feedback. Center 12 had interpreted the terminology “cluster” differently and instead reported the Nextclade assignment (10); center 14 in turn deemed samples NGS1 and NGS4 to belong to a single cluster. While they share an ancestor, most other laboratories deemed them sufficiently different to assign them to two separate clusters. In fact, they differ in 27 SNPs, whereas the other true clusters (clusters B, C, and E in Table 3) had 0 to 1 SNPs between genomes. This highlights that there is a certain element of subjectivity in data interpretation when lacking clear definitions as well as the need to clarify the objective of the task (in this case the assessment of transmission clusters rather than simply related sequences in a phylogenetic tree).

An important factor for routine sequencing is cost. In general, the amplicon-based protocols used in this study consist of a reverse transcription step, an amplification step, library preparation, and sequencing. As the first two steps are mostly the same for different sequencing technologies, cost is driven mainly by library preparation and sequencing itself. Here, Oxford Nanopore sequencing allows faster data generation due to real-time base calling, while sequencing on an Illumina machine typically takes slightly more than a day (11). Cost-wise, the price per sample will decrease with increasing throughput. But the many library preparation kits available as well as the wide range of sequencing machines used here (Table 1) make comparisons between the centers difficult.

All protocols used by the participating centers in this EQA used amplicon-based sequencing, and primer bias can have an influence on sequencing accuracy. Here, primer sets vary between laboratories (Table 1). For the Artic v3 primers (which are public), we find no apparent bias in the data reported here compared to the other primer panels. However, centers 7 and 8 used the same primer panel but did not detect the variant G21255C in samples NGS3, -6, and -8 (Fig. 1C, F, and H). This SNP is present in almost all representatives of lineage B.1.177 (12). Whether this failure in detection is truly due to primer bias cannot be conclusively answered, however, as commercial primer sequences are often not public. A possibility to deal with this issue bioinformatically is to trim primer sequences prior to assembly. Nevertheless, primer bias is a real issue if it leads to dropouts. Fortunately, this is actively monitored by the community. For example, dropouts of the Artic v3 panel have been reported, especially for Beta and Delta variants. For this reason, a new primer panel has been developed to avoid high-frequency variant sites in the newer lineages (13).

### Factors not assessed in this pilot EQA.

This pilot EQA focused on reporting findings related to consensus genome sequences but did not include minority variant reporting. Center 15 reported issues with contamination for sample NGS8, yet lineage and cluster assignments were successful as the key sites were not affected. However, some contamination spilled over into the consensus genome as evidenced by a number of wrong variant calls (Fig. S1H). Similarly, some laboratories reported mixed loci as SNPs in their reports, although we were mostly interested in fixed changes. Differentiating between contamination and true, albeit rare, mixed infections or possible in-host evolution can be very difficult, especially in a clinical setting with high sample throughput. Assessment of contamination and analysis of minority variants would allow the provision of more detailed feedback to the laboratories. Contamination, for example, would likely be an isolated event for a center, resulting in mixed sites, while a true mixture would be prevalent across all centers. At the same time, it would offer an interesting analytical challenge, particularly if samples with true mixed infections were sent to participants.

### Conclusion and lessons learned.

The first ESGMD-SSM pilot EQA of SARS-CoV-2 sequencing was overall a success. Most centers generated whole-genome sequences and correctly identified all lineages and clusters. Additionally, there was a consensus regarding the majority of called SNPs despite the strong effect that missing data and unreported deletions (although present in the data) had on the scores of some. This suggests an overall high quality in each participating center. The standardized reporting of important variations in the genome should be the focus of improvement for some bioinformatic pipelines. The most critical aspect was coverage across the genome, which correlated with correct lineage and cluster assignments.

For a follow-up EQA, the variables and format of the variables to document have to be more clearly defined. Moreover, minority variants should be included to some degree from samples with mixed infections. Information on primer sets for amplicon-based methods should be carefully recorded, especially in light of new virus lineages. Instead of culture supernatants, it might also be of interest to include primary patient samples diluted in a clinical collection matrix as well as an empty control. Finally, to trigger a discussion on cluster definition, samples with high similarity but 2 to 5 SNP differences could also be included.

The COVID-19 pandemic required a rapid global laboratory response involving the development and rollout of new diagnostic assays and diagnostic platforms on an unprecedented scale. In response to the emergence and spread of virus variants of concern, WGS is increasingly being utilized not only for surveillance but also for diagnostic purposes, thus necessitating the rapid deployment and sharing of quality assurance schemes. This EQA pilot provides proof of feasibility for the development and operationalization of an EQA for WGS in a pandemic context, and lessons learned from its design, delivery, and results should inform future pandemic preparedness.

## References

[1] European Centre for Disease Prevention and Control. 2021. Sequencing of SARS-CoV-2: first update. European Centre for Disease Prevention and Control, Solna, Sweden. https://www.ecdc.europa.eu/en/publications-data/sequencing-sars-cov-2.

[2] Karmarkar EN, Blanco I, Amornkul PN, DuBois A, Deng X, Moonan PK, Rubenstein BL, Miller DA, Kennedy I, Yu J, Dauterman JP, Ongpin M, Hathaway W, Hoo L, Trammell S, Dosunmu EF, Yu G, Khwaja Z, Lu W, Talai NZ, Jain S, Louie JK, Philip SS, Federman S, Masinde G, Wadford DA, Bobba N, Stoltey J, Smith A, Epson E, Chiu CY, Bennett AS, Vasquez AM, Williams T. 2021. Timely intervention and control of a novel coronavirus (COVID-19) outbreak at a large skilled nursing facility—San Francisco, California, 2020. Infect Control Hosp Epidemiol 42:1173–1180. 10.1017/ice.2020.1375. 33308357 PMC8144818

[3] Pillay S, Giandhari J, Tegally H, Wilkinson E, Chimukangara B, Lessells R, Moosa Y, Mattison S, Gazy I, Fish M, Singh L, Khanyile KS, San JE, Fonseca V, Giovanetti M, Alcantara LC, Jr, de Oliveira T. 2020. Whole genome sequencing of SARS-CoV-2: adapting Illumina protocols for quick and accurate outbreak investigation during a pandemic. Genes (Basel) 11:949. 10.3390/genes11080949. PMC7464704 32824573

[4] Stange M, Mari A, Roloff T, Seth-Smith HMB, Schweitzer M, Brunner M, Leuzinger K, Søgaard KK, Gensch A, Tschudin-Sutter S, Fuchs S, Bielicki J, Pargger H, Siegemund M, Nickel CH, Bingisser R, Osthoff M, Bassetti S, Schneider-Sliwa R, Battegay M, Hirsch HH, Egli A. 2021. SARS-CoV-2 outbreak in a tri-national urban area is dominated by a B.1 lineage variant linked to a mass gathering event. PLoS Pathog 17:e1009374. 10.1371/journal.ppat.1009374. 33740028 PMC8011817

[5] Brüningk SC, Klatt J, Stange M, Mari A, Brunner M, Roloff T-C, Seth-Smith HMB, Schweitzer M, Leuzinger K, Søgaard KK, Torres DA, Gensch A, Schlotterbeck A-K, Nickel CH, Ritz N, Heininger U, Bielicki J, Rentsch K, Fuchs S, Bingisser R, Siegemund M, Pargger H, Ciardo D, Dubuis O, Buser A, Tschudin-Sutter S, Battegay M, Schneider-Sliwa R, Borgwardt KM, Hirsch HH, Egli A. 2020. Determinants of SARS-CoV-2 transmission to guide vaccination strategy in a city. medRxiv 10.1101/2020.12.15.20248130. PMC8927799 35310621

[6] Junier T, Huber M, Schmutz S, Kufner V, Zagordi O, Neuenschwander S, Ramette A, Kubacki J, Bachofen C, Qi W, Laubscher F, Cordey S, Kaiser L, Beuret C, Barbié V, Fellay J, Lebrand A. 2019. Viral metagenomics in the clinical realm: lessons learned from a Swiss-wide ring trial. Genes (Basel) 10:655. 10.3390/genes10090655. PMC6770386 31466373

[7] Dylus D, Pillonel T, Opota O, Wüthrich D, Seth-Smith HMB, Egli A, Leo S, Lazarevic V, Schrenzel J, Laurent S, Bertelli C, Blanc DS, Neuenschwander S, Ramette A, Falquet L, Imkamp F, Keller PM, Kahles A, Oberhaensli S, Barbié V, Dessimoz C, Greub G, Lebrand A. 2020. NGS-based S. aureus typing and outbreak analysis in clinical microbiology laboratories: lessons learned from a Swiss-wide proficiency test. Front Microbiol 11:591093. 10.3389/fmicb.2020.591093. 33424794 PMC7793906

[8] Rambaut A, Holmes EC, O’Toole Á, Hill V, McCrone JT, Ruis C, Du Plessis L, Pybus OG. 2020. A dynamic nomenclature proposal for SARS-CoV-2 lineages to assist genomic epidemiology. Nat Microbiol 5:1403–1407. 10.1038/s41564-020-0770-5. 32669681 PMC7610519

[9] Leuzinger K, Gosert R, Søgaard KK, Naegele K, Bielicki J, Roloff T, Bingisser R, Nickel CH, Khanna N, Sutter ST, Widmer AF, Rentsch K, Pargger H, Siegemund M, Stolz D, Tamm M, Bassetti S, Osthoff M, Battegay M, Egli A, Hirsch HH. 2021. Epidemiology and precision of SARS-CoV-2 detection following lockdown and relaxation measures. J Med Virol 93:2374–2384. 10.1002/jmv.26731. 33314153

[10] Aksamentov I, Neher R. 2020. Nextclade. https://github.com/nextstrain/nextclade.

[11] Hourdel V, Kwasiborski A, Balière C, Matheus S, Batéjat CF, Manuguerra J-C, Vanhomwegen J, Caro V. 2020. Rapid genomic characterization of SARS-CoV-2 by direct amplicon-based sequencing through comparison of MinION and Illumina iSeq100 system. Front Microbiol 11:571328. 10.3389/fmicb.2020.571328. 33101244 PMC7546329

[12] Hodcroft EB, Zuber M, Nadeau S, Vaughan TG, Crawford KHD, Althaus CL, Reichmuth ML, Bowen JE, Walls AC, Corti D, Bloom JD, Veesler D, Mateo D, Hernando A, Comas I, González-Candelas F, SeqCOVID-SPAIN Consortium, Stadler T, Neher RA. 2021. Spread of a SARS-CoV-2 variant through Europe in the summer of 2020. Nature 595:707–712. 10.1038/s41586-021-03677-y. 34098568

[13] Davis JJ, Long SW, Christensen PA, Olsen RJ, Olson R, Shukla M, Subedi S, Stevens R, Musser JM. 2021. Analysis of the ARTIC version 3 and version 4 SARS-CoV-2 primers and their impact on the detection of the G142D amino acid substitution in the spike protein. bioRxiv 10.1101/2021.09.27.461949. PMC8653831 34878296

[14] Schmutz S, Huber M, Zaheri M, Zagordi O. 2021. SmaltAlign. https://github.com/medvir/SmaltAlign.

[15] Loman N, Rowe W, Rambaut A. 2020. nCoV-2019 novel coronavirus bioinformatics protocol. https://artic.network/ncov-2019/ncov2019-bioinformatics-sop.html.

[16] Maier W, Bray S, van den Beek M, Bouvier D, Coraor N, Miladi M, Singh B, De Argila JR, Baker D, Roach N, Gladman S, Coppens F, Martin DP, Lonie A, Grüning B, Kosakovsky Pond SL, Nekrutenko A. 2021. Freely accessible ready to use global infrastructure for SARS-CoV-2 monitoring. bioRxiv 10.1101/2021.03.25.437046. PMC8845060 34588690

[17] Jalili V, Afgan E, Gu Q, Clements D, Blankenberg D, Goecks J, Taylor J, Nekrutenko A. 2020. The Galaxy platform for accessible, reproducible and collaborative biomedical analyses: 2020 update. Nucleic Acids Res 48:W395–W402. 10.1093/nar/gkaa434. 32479607 PMC7319590

[18] Le G, Veer B, Jamin C. 2021. MACOVID. https://github.com/MUMC-MEDMIC/MACOVID.

[19] Oude Munnink BB, Nieuwenhuijse DF, Stein M, O’Toole Á, Haverkate M, Mollers M, Kamga SK, Schapendonk C, Pronk M, Lexmond P, van der Linden A, Bestebroer T, Chestakova I, Overmars RJ, van Nieuwkoop S, Molenkamp R, van der Eijk AA, GeurtsvanKessel C, Vennema H, Meijer A, Rambaut A, van Dissel J, Sikkema RS, Timen A, Koopmans M, Dutch-Covid-19 Response Team. 2020. Rapid SARS-CoV-2 whole-genome sequencing and analysis for informed public health decision-making in the Netherlands. Nat Med 26:1405–1410. 10.1038/s41591-020-0997-y. 32678356

[20] Corman VM, Landt O, Kaiser M, Molenkamp R, Meijer A, Chu DKW, Bleicker T, Brünink S, Schneider J, Schmidt ML, Mulders DGJC, Haagmans BL, van der Veer B, van den Brink S, Wijsman L, Goderski G, Romette J-L, Ellis J, Zambon M, Peiris M, Goossens H, Reusken C, Koopmans MPG, Drosten C. 2020. Detection of 2019 novel coronavirus (2019-nCoV) by real-time RT-PCR. Euro Surveill 25:2000045. 10.2807/1560-7917.ES.2020.25.3.2000045. PMC6988269 31992387

[21] Coolen JPM, Wolters F, Tostmann A, van Groningen LFJ, Bleeker-Rovers CP, Tan ECTH, van der Geest-Blankert N, Hautvast JLA, Hopman J, Wertheim HFL, Rahamat-Langendoen JC, Storch M, Melchers WJG. 2021. SARS-CoV-2 whole-genome sequencing using reverse complement PCR: for easy, fast and accurate outbreak and variant analysis. J Clin Virol 144:104993. 10.1016/j.jcv.2021.104993. 34619382 PMC8487099

[22] Coolen J. 2021. Easyseq. https://github.com/JordyCoolen/easyseq_covid19.

[23] Hetland M. 2021. SusCovONT. https://github.com/marithetland/susCovONT.

[24] Health 2030 Genome Center. 2021. SARS-CoV-2_pipeline. https://github.com/health2030genomecenter/SARS-CoV-2_pipeline.

[25] Grubaugh ND, Gangavarapu K, Quick J, Matteson NL, De Jesus JG, Main BJ, Tan AL, Paul LM, Brackney DE, Grewal S, Gurfield N, Van Rompay KKA, Isern S, Michael SF, Coffey LL, Loman NJ, Andersen KG. 2019. An amplicon-based sequencing framework for accurately measuring intrahost virus diversity using PrimalSeq and iVar. Genome Biol 20:8. 10.1186/s13059-018-1618-7. 30621750 PMC6325816

[26] Pagès H, Aboyoun P, Gentleman R, DebRoy S. 2017. Biostrings: efficient manipulation of biological strings. R package version 2.46.0.

[27] Mari A, Roloff T, Stange M, Søgaard KK, Asllanaj E, Tauriello G, Alexander LT, Schweitzer M, Leuzinger K, Gensch A, Martinez AE, Bielicki J, Pargger H, Siegemund M, Nickel CH, Bingisser R, Osthoff M, Bassetti S, Sendi P, Battegay M, Marzolini C, Seth-Smith HMB, Schwede T, Hirsch HH, Egli A. 2021. Global genomic analysis of SARS-CoV-2 RNA dependent RNA polymerase evolution and antiviral drug resistance. Microorganisms 9:1094. 10.3390/microorganisms9051094. 34069681 PMC8160703

[28] Mari A. 2021. COVGAP. https://github.com/appliedmicrobiologyresearch/covgap.

[29] metagenlab. 2021. GENCOV. https://github.com/metagenlab/GENCOV.

