# Supplementary material for: External Quality Assessment of SARS-CoV-2 Sequencing: an ESGMD-SSM Pilot Trial across 15 European Laboratories
Source: J Clin Microbiol. 2022 Jan 19;60(1):e01698-21. doi: 10.1128/JCM.01698-21 (PMC8769736; doi:10.1128/JCM.01698-21)
Supplement: Supplemental file 1 — Text S1. Download JCM.01698-21-s0001.pdf, PDF file, 812 KB [file jcm.01698-21-s0001.pdf]

Supplemental Figures

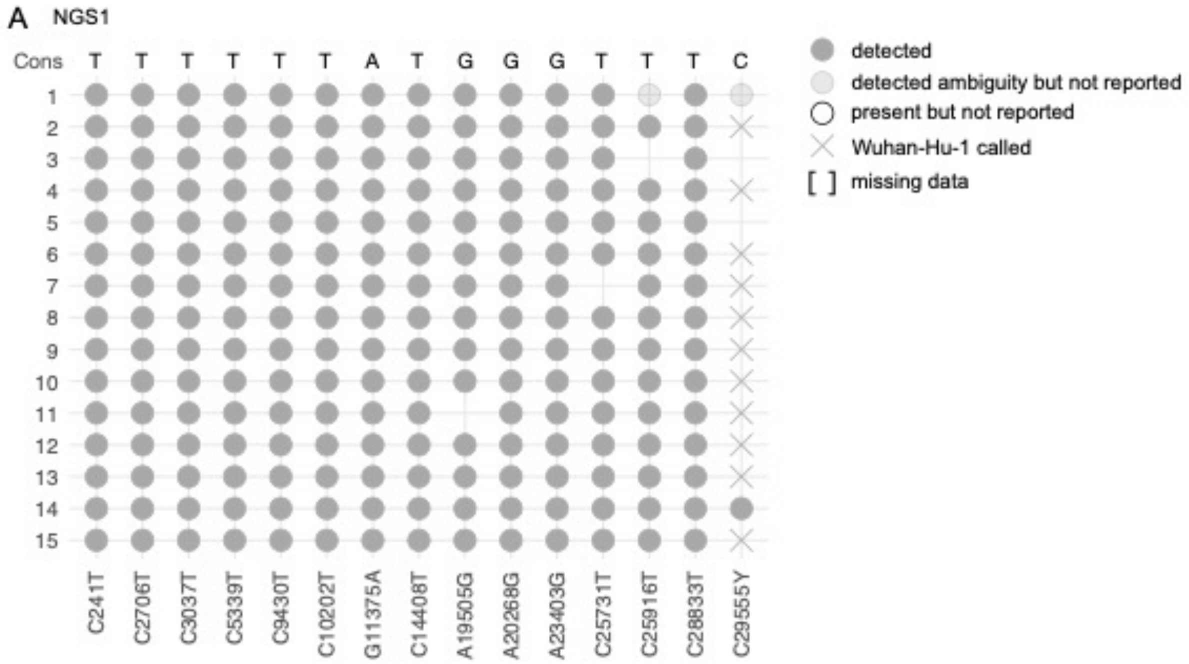

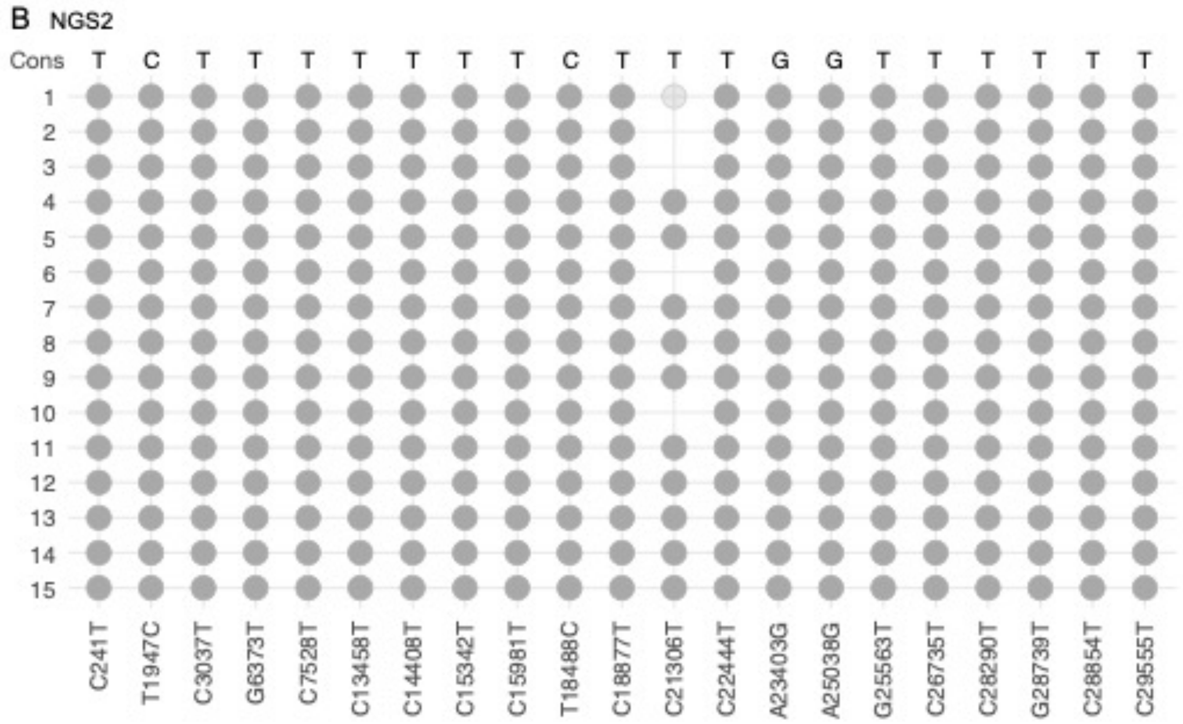

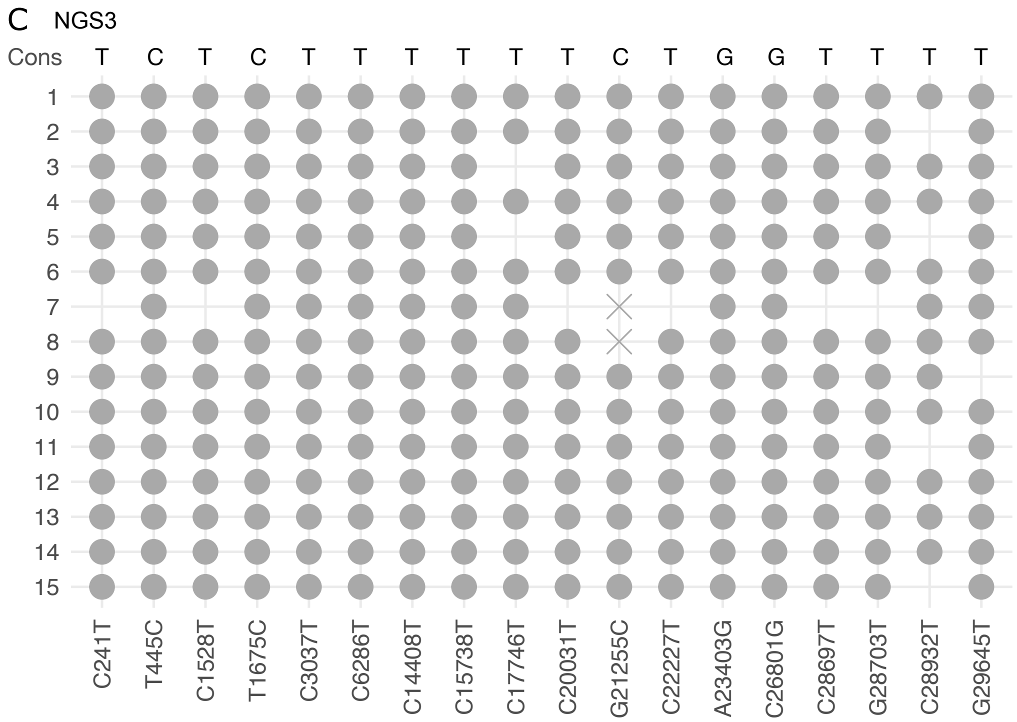
C NGS3
Cons T C T C T T T T T T C T G G T T T T
1 2 3 4 5 6 7 8 9 10 11 12 13 14 15
C241T T445C C1528T T1675C C3037T C6286T C14408T C15738T C17746T C20031T G21255C C22227T A23403G C26801G C28697T G28703T C28932T G29645T

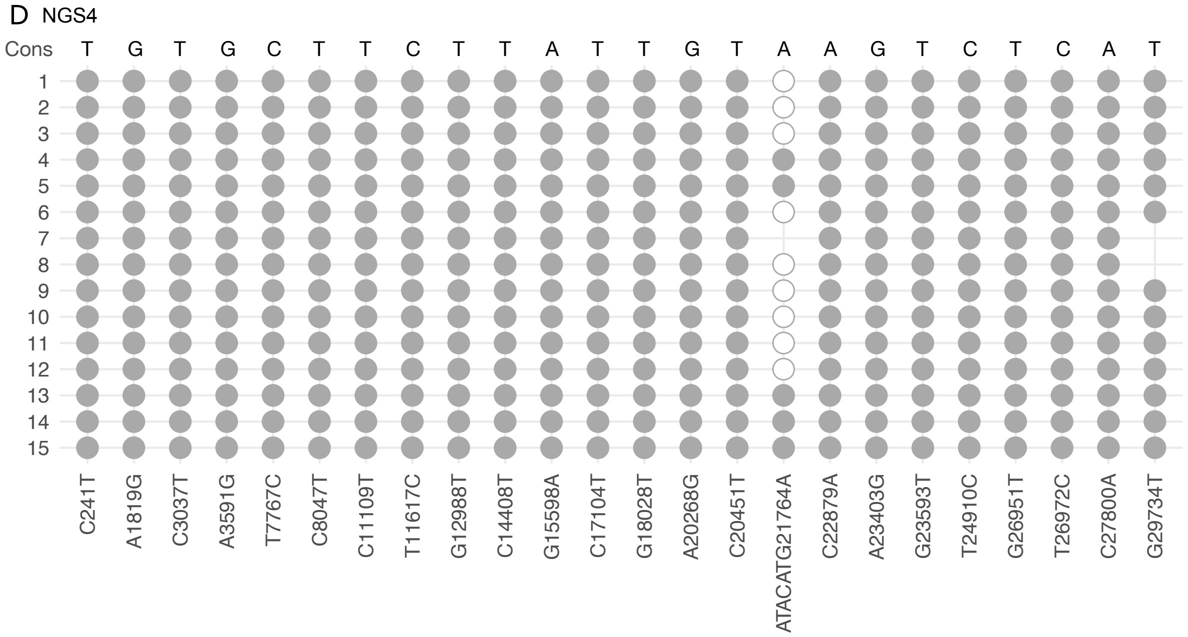
D NGS4
Cons T G T G C T T C T T A T T G T A A G T C T C A T
1 2 3 4 5 6 7 8 9 10 11 12 13 14 15
C241T A1819G C3037T A3591G T7767C C8047T C11109T T11617C G12988T C14408T G15598A C17104T G18028T A20268G C20451T ATACATG21764A C22879A A23403G G23593T T24910C G26951T T26972C C27800A G29734T

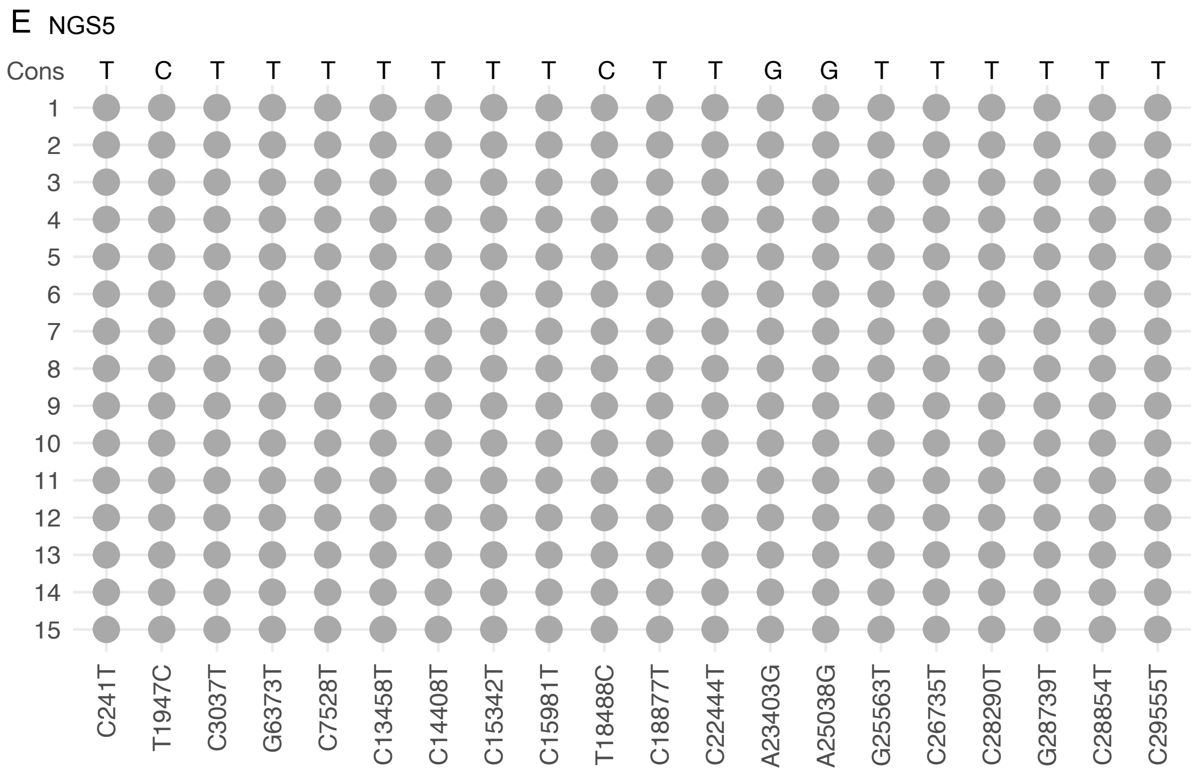
E NGS5
Cons
T C T T T T T T T C T T G G T T T T T T
1
2
3
4
5
6
7
8
9
10
11
12
13
14
15
C241T
T1947C
C3037T
G6373T
C7528T
C13458T
C14408T
C15342T
C15981T
T18488C
C18877T
C22444T
A23403G
A25038G
G25563T
C26735T
C28290T
G28739T
C28854T
C29555T

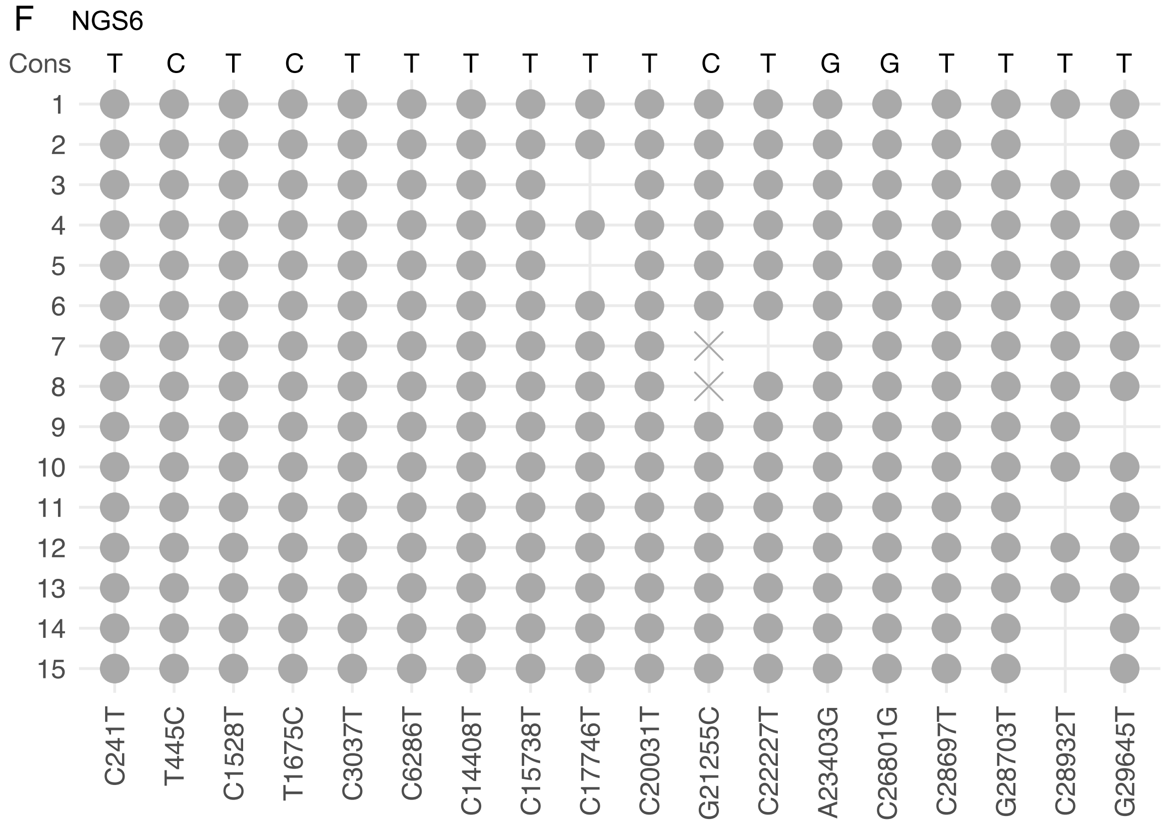
F NGS6
Cons
T C T C T T T T T T C T G G T T T T
1
2
3
4
5
6
7
8
9
10
11
12
13
14
15
C241T
T445C
C1528T
T1675C
C3037T
C6286T
C14408T
C15738T
C17746T
C20031T
G21255C
C22227T
A23403G
C26801G
C28697T
G28703T
C28932T
G29645T

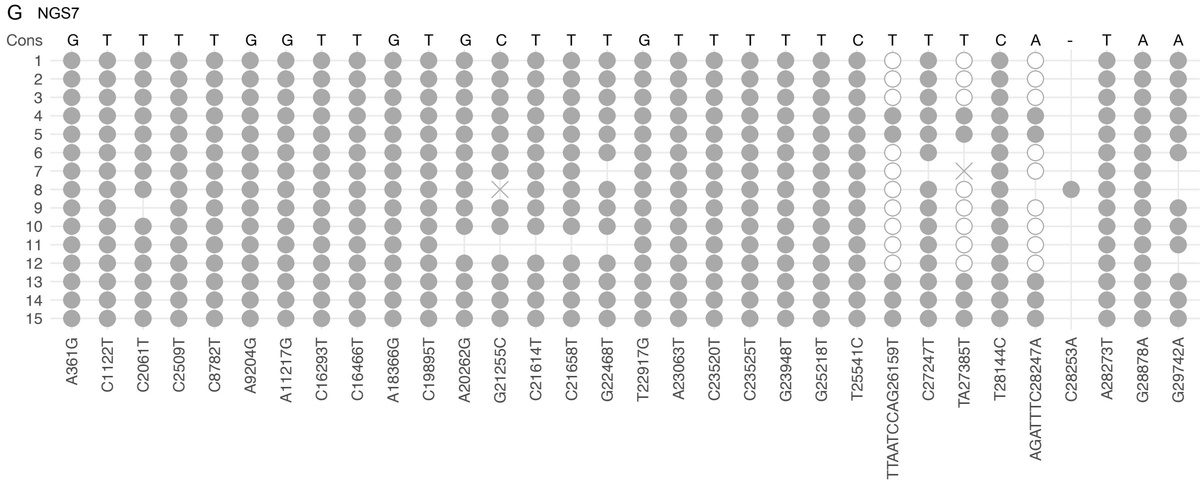
G NGS7
Cons G T T T T G G T T G T G C T T T G T T T T T C T T T C A - T A A
1 2 3 4 5 6 7 8 9 10 11 12 13 14 15
A361G C1122T C2061T C2509T C8782T A9204G A11217G C16293T C16466T A18366G C19895T A20262G G21255C C21614T C21658T G22468T T22917G A23063T C23520T C23525T G23948T G25218T T25541C TTAATCCAG26159T C27247T TA27385T T28144C AGATTTC28247A C28253A A28273T G28878A G29742A

9

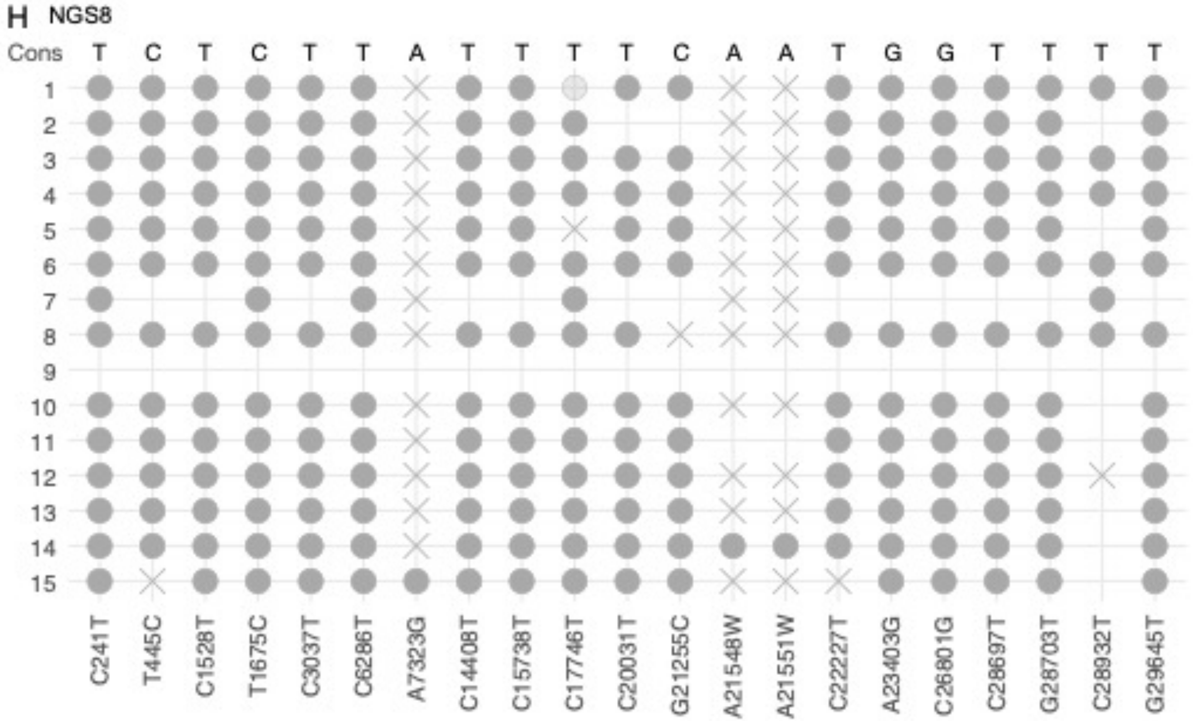
H NGS8
Cons T C T C T T A T T T T C A A T G G T T T T
1 2 3 4 5 6 7 8 9 10 11 12 13 14 15
C241T T445C C1528T T1675C C3037T C6286T A7323G C14408T C15738T C17746T C20031T G21255C A21548W A21551W C22227T A23403G C26801G C28697T G28703T C28932T G29645T

10

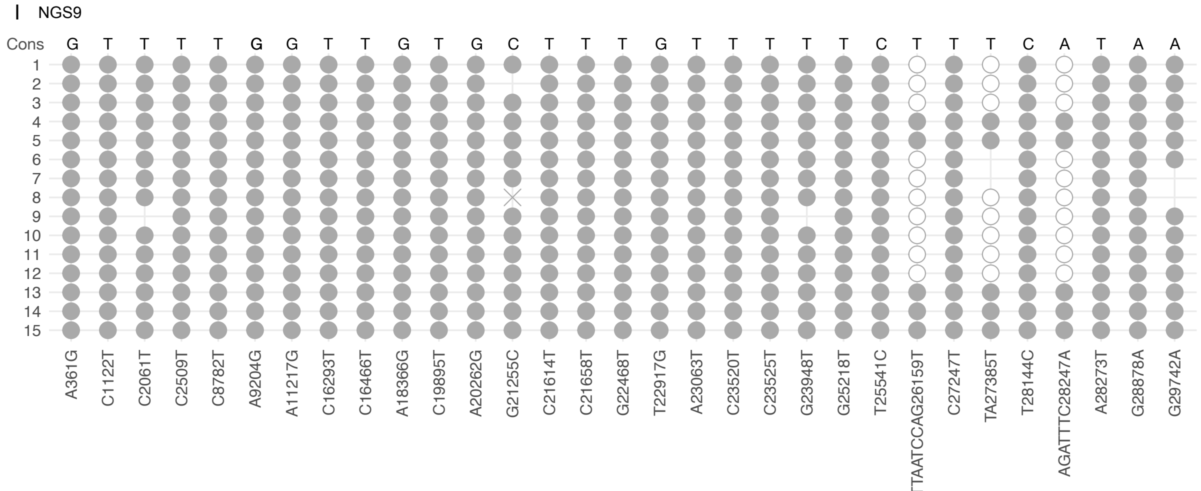
I NGS9
Cons G T T T T G G T T G T G C T T T G T T T T T C T T T C A T A A
1 2 3 4 5 6 7 8 9 10 11 12 13 14 15
A361G C1122T C2061T C2509T C8782T A9204G A11217G C16293T C16466T A18366G C19895T A20262G G21255C C21614T C21658T G22468T T22917G A23063T C23520T C23525T G23948T G25218T T25541C TTAATCCAG26159T C27247T TA27385T T28144C AGATTTC28247A A28273T G28878A G29742A

11

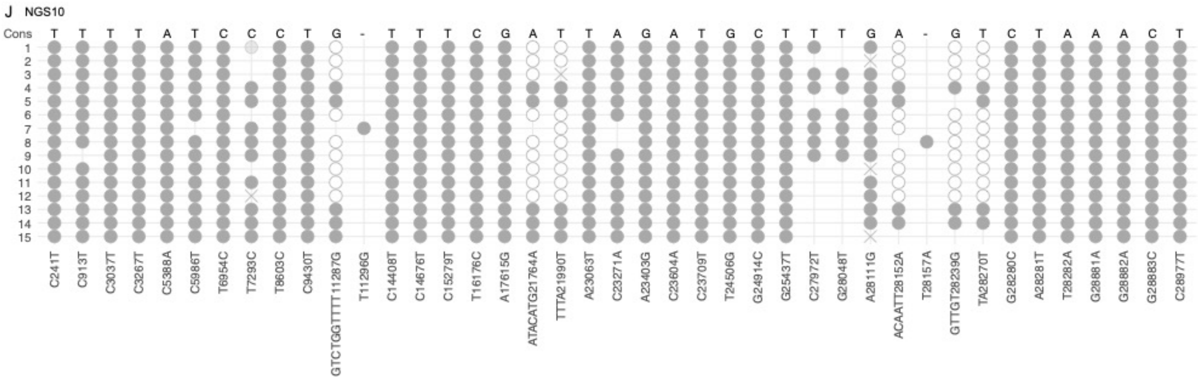

Suppl. figure S1: A-J Presence and absence of SNPs and Indels per sample. On the x-axis, all variations that were specifically reported by the centres are listed. On the y-axis are the centres. A dark grey filled circle means the respective SNP was reported. No symbol means the genome sequence has an N at that position. A cross indicates that instead of the SNP, the reference position was called; this can either be because the SNP is not true or because the base call is wrong. Additionally, sometimes ambiguous sites were reported as SNPs or are present in the consensus genome at the position of a reported SNP. If such a position was found in the sequence (but not reported) a less opaque filled circle is shown. Lastly, some centres did not report deletions. If these non-reported deletions were nevertheless present in the data, they are indicated with a white-filled circle.

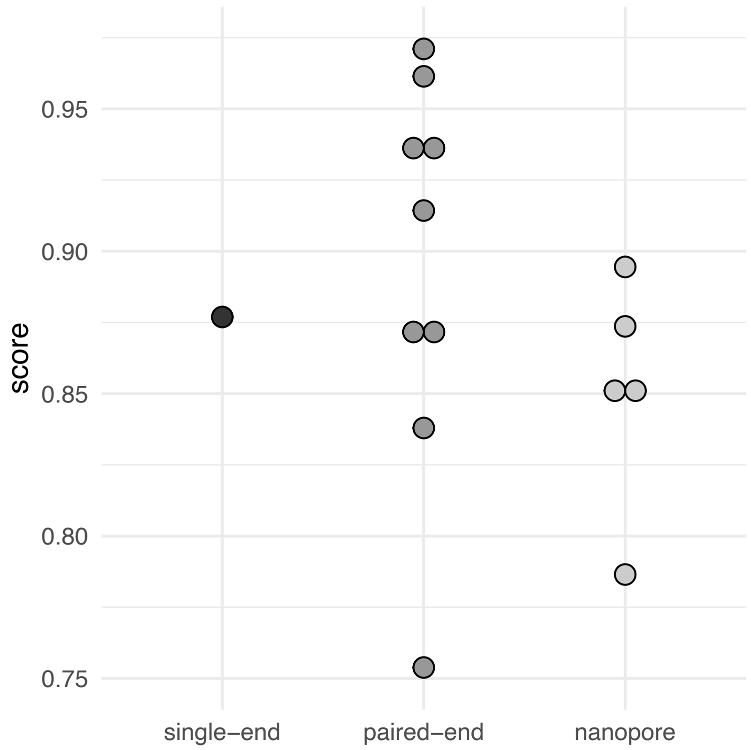

Suppl. figure S2: Mean variant calling score per lab depending on the sequencing methods used.

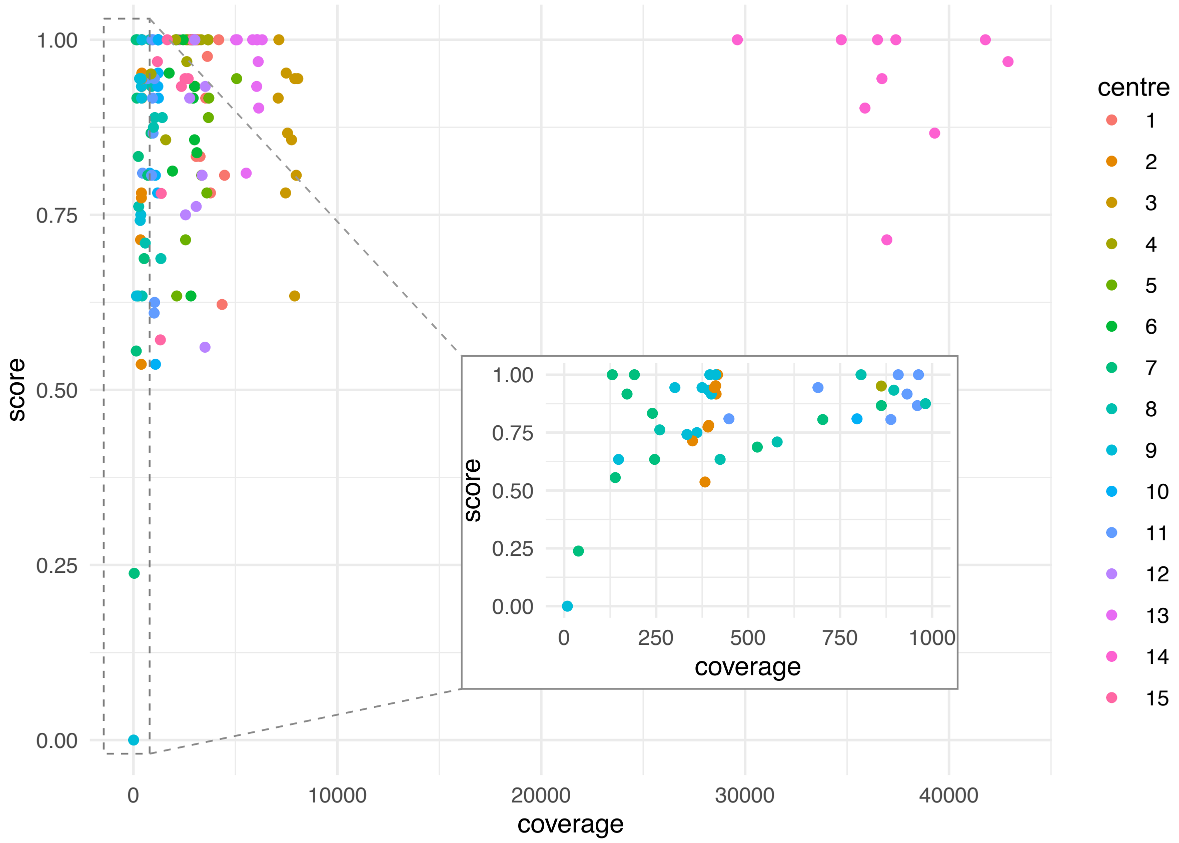

Suppl. figure S3: Variant calling score for each sample and centre depending on the mean coverage.
