# Supplementary material for: External Quality Assessment of SARS-CoV-2 Sequencing: an ESGMD-SSM Pilot Trial across 15 European Laboratories
Source: J Clin Microbiol. 2022 Jan 19;60(1):e01698-21. doi: 10.1128/JCM.01698-21 (PMC8769736; doi:10.1128/JCM.01698-21)
Supplement: Supplemental file 2 — Text S2. Download JCM.01698-21-s0002.pdf, PDF file, 154 KB [file jcm.01698-21-s0002.pdf]

Supplemental Methods

Centre 1

SARS-CoV-2 whole-genome sequencing was performed according to the nCoV-2019 sequencing protocol v3 (LoCost) V.3 (1). Briefly, total nucleic acids were extracted followed by reverse transcription with random hexamers using LunaScript RT SuperMix Kit (NEB). The generated cDNA was used as input for two pools of overlapping PCR reactions (ca. 400nt each) spanning the viral genome using Q5 Hot Start High-Fidelity 2X Master Mix (NEB). Amplicons were pooled per patient before NexteraXT library preparation and sequencing on an Illumina MiSeq for 1 × 151 cycles. To generate SARS-CoV-2 consensus sequences, reads were iteratively aligned using SmaltAlign (2). Clusters were determined manually based on phylogenetic analysis.

Centre 2

A typical Nanopore sequencing library consisted of the pooling of PCR amplicons generated according to the ARTIC v3 protocol (3), which generates 400 bp amplicons that overlap by approximately 20 bp. Library preparation was performed with SQK-LSK109 (Oxford Nanopore Technologies, Oxford, UK) according to the ONT "PCR tiling of COVID-19 virus" (version: PTC_9096_v109_revE_06Feb2020, last update: 26/03/2020). Reagents, quality control and flow cell preparation were done as described previously (4,5). ONT sequencing was performed on a GridION X5 instrument (Oxford Nanopore Technologies) with real-time basecalling enabled (ont-guppy-for-gridion v.4.2.3; fast basecalling mode). Sequencing runs

were terminated after production of at least 100,000 reads per sample. Bioinformatic analyses followed the workflow described (3) using artic version 1.1.3. Consensus sequences were generated using medaka (6) and bcftools (7). For cluster determination, the consensus sequences were aligned using muscle (v3.8.1551, options -maxiters 1 -diags), and the number of nucleotide differences between each sequence pair was calculated with R (version #.6.0) using the R libraries seqinr and dplyr. Cluster definition was set as no SNV difference between any sequences in a given cluster.

Centre 3

The RNA of the samples was extracted with the Maxwell RSC Viral TNA kit and tested with our inhouse-house SARS-CoV-2 assay. The reverse transcription was done with the LunaScript RT Super Mix (NEB), followed by amplification of the SARS-CoV-2 genome according to the amplicon sequencing strategy of the ARCTIC protocol with re-balanced V.3 primers. Library construction was performed with the Illumina DNA Prep (M) kit according to the manufacturer’s instructions. After quantification, an equal amount of each library was pooled and sequenced on an Illumina MiSeq with 300 cycles and v2 chemistry. The bioinformatics analysis was done with the virSEAK pipeline (v2.0.11; JSI). The discrimination into the different clusters was done manually according to the designated Pango lineage.

Centre 4

RNA from nasopharyngeal or mouth swabs collected in COPAN UTM™ liquid (3.5 ml) were extracted on a MagNA Pure 96 instrument (Roche, Basel, Switzerland). All samples were

processed with the CleanPlex SARS-CoV-2 15 Panel and CleanPlex Dual Indexed (Paragon Genomics #918011) according to manufacturer's protocol. PCR products were analyzed using a Fragment Analyzer, « Standard Sensitivity NGS » (AATI, ref. DNF-473), and DNA was quantified with Qubit Standard Sensitivity dsDNA kit (Invitrogen, ref. Q32853). All samples were sequenced using paired-end 2x150bp MiSeq Illumina protocol (San Diego, USA). Sequence reads were processed using GENCOV (8), a modified version of CoVpipe (9). Briefly, reads were filtered with fastp (10) and mapped on SARS-CoV-2 reference genome NC_045512.2 with bwa (11). Qualimap (12) was used to evaluate the alignment and primer sequences from CleanPlex® panel were trimmed with fgbio (13). Variant calling was performed with freebayes (14) (Parameters: --min-alternate-fraction 0.1 --min-coverage 10 --min-alternate-count 9). Putative variants were filtered with bcftools (15) based on mean mapping quality (MQM > 40), variant quality (QUAL >10) and an alternate frequency of at least 70%. The consensus sequence generated with bcftools was assigned to SARS-Cov-2 lineages with pangolin (16).

Centre 5

Whole genome sequencing. cDNA was produced from extracted RNA using random hexamer primers and Superscript III (ThermoFisher) followed by a PCR tiling the entire SARS-CoV-2 genome (ARTIC V3 primer sets; (17)). This produced 400 bp long, overlapping amplicons that were subsequently used to prepare the sequencing library. Briefly, the amplicons were cleaned with AMPure magnetic beads (Beckman Coulter). Afterwards the QIAseq FX DNA Library Kit (Qiagen) was used to prepare indexed paired end libraries for Illumina sequencing. Normalized and pooled sequencing libraries were denatured with 0.2 N

NaOH. This 8 pM library was sequenced on an Illumina MiSeq instrument using the 300-cycle MiSeq Reagent Kit v2.

Bioinformatics. The de-multiplexed raw reads were subjected to a custom Galaxy pipeline (18,19). The raw reads were pre-processed with fastp (v.0.20.1) (10) and mapped to the SARS-CoV-2 Wuhan-Hu-1 reference genome (Genbank: NC_045512) using BWA-MEM (v.0.7.17) (20). For datasets, which were produced with the ARTIC v3 protocol, primer sequences were trimmed with ivar trim (v1.9) (21). Variants (SNPs and INDELs) were called with the ultrasensitive variant caller LoFreq (v2.1.5) (22) demanding a minimum base quality of 30 and a coverage of at least 5-fold. Afterwards, the called variants were filtered based on a minimum variant frequency of 10 % and on the support of strand bias. The effects of the mutations were automatically annotated in the vcf files with SnpEff (v.4.3.1) (23). Finally, consensus sequences were constructed by bcftools (v.1.1.0) (24). Regions with low coverage >5x or variant frequencies between 30 and 70 % were masked with Ns. The variant frequencies (>10%) of the nucleotide substitutions of the respective samples were matched in a matrix and clusters were determined by hierarchical clusterin (ward.D2) using the R package hclust. The script is available on GitHub (25)and was implemented on usegalaxy.eu.

Centre 6

Sequencing of SARS-CoV-2-positive samples

Samples were stored at -80 degrees Celsius until RNA was isolated for sequencing. For RNA extraction, 90 µl of sample was mixed with 90 µl of Chemagic Viral Lysis Buffer (Perkin-Elmer), followed by extraction using the MagNA Pure 96 DNA and Viral NA Small Volume

Kit 96 (Roche, Germany) on the MagNA Pure 96 system (Roche, Germany), without the addition of an internal extraction control.

Sequencing was performed using the PCR tiling of SARS-CoV-2 virus with Native Barcoding Expansion 96 (EXP-NBD196) protocol (Version: PTCN_9103_v109_revH_13Jul2020) of Oxford Nanopore technologies, with minor modifications and using the primers previously published by Oude Munnink et al. (26). Briefly, the only modifications were extending the barcode and adaptor ligation steps up to 60 min and loading 48 samples per flow cell.

Bioinformatic analysis was performed using an in-house developed pipeline MACOVID that is based on Artic v1.1.3. In brief, short and obvious chimeric reads are filtered with Cutadapt v2.5. The filtered reads were mapped to the reference genome MN908947.3 with Minimap2 v2.17 and quality checked with "align_trim" function of Artic v1.1.3. Mapped reads were split per primer pool using Samtools v1.9 and a consensus was created per primer pool with Medaka v1.0.3. Variants were called using Medaka v1.0.3 and Longshot v0.4.1. Low coverage regions (<30x) were masked with "artic_make_depth_mask" function of Artic v1.1.3. A preconsensus was made with "artic_mask" and the final consensus sequence was made with bcftools v1.10.2. Documentation and source code are available from (27) under MIT license. The consensus sequences were used to construct a phylogenetic tree with the ncov pipeline v3 of nextstrain. Samples were considered to be part of the same cluster of there are <= 2 SNPs difference. Pangolin lineages were assigned were assigned using the Pangolin COVID-19 Lineage Assigner web application on https://pangolin.cog-uk.io/.

Centre 7

Nucleic acid was extracted from 200 ul sample and eluted in 100 ul buffer using a MagNa Pure 96 instrument (Roche Diagnostics). Ten microliters extract was added to the RT-PCR assay for SARS-CoV-2 E-gene detection as described by Corman et al. (28) and performed on a CFX96 PCR instrument (Bio-Rad): 50˚C for 5 min, followed by 95˚C for 20 s and then 45 cycles of 95˚C for 15 s, 55˚C for 10 s, and 72˚C for 50 s.

Whole genome sequencing (WGS) was performed using the EasySeq RC-PCR SARS-CoV-2 WGS kit (NimaGen BV). A detailed description of the technology has recently been described by Coolen et al, 2020 (29). Bidirectional sequencing of the SARS-CoV-2 amplicons was performed using the MiniSeq platform (Illumina), with fastQ-formatted sequences being extracted from the MiniSeq machine and processed further using different bioinformatic tools. First, quality filtering of reads, including trimming of primer sequences, was performed using Trimmomatic (version 3) with the following settings: LEADING:3; TRAILING:3; SLINDINGWINDOW:4:15; HEADCROP:32; MINLEN:40. Then, reads were mapped with Bowtie2 (version 2.3.4, settings --local --qc-filter --quiet) to the NC_045512.2 SARS-CoV-2 reference strain and further analyzed using the default settings of Samtools (version 1.7). The sequence read depth was calculated using the IGV tool (version 2.3.98, settings: -w 1). Values of read depth obtained for each position (NTs or indels) for all samples were filtered using 0.5 as a minimum frequency of SNPs relative to the total depth at this position, so S/VNPs with frequency of <0.5 were ignored. Positions with a read depth of <10 reads were also ignored and implemented in sequences as gaps and filled with Ns. A list of SNPs found compared to NC_045512.2 was generated after uploading the consensus sequences to Nextclade (version 0.14.2) and downloading the resulting CSV file. Finally, sequences with >=50% non-gap positions were used for building a phylogenetic tree.

Phylogenetic analysis of the data was done with Nextstrain (version 1.16.5) and a maximum likelihood tree was built with IQ-TREE (settings: -ninit 2 -n 2 -me 0.05 -nt 1). Results of the analysis were represented as Auspice v2 JSON files. Clusters were identified by having no more than three SNP difference.

Centre 8

DNA sequencing and analysis was performed similar to method described in (29). In short: cDNA-synthesis was performed using Multiscribe RT (Applied Biosystems, CA, USA). Whole genome sequencing (WGS) was performed using EasySeqTM RC-PCR SARS-CoV-2 version 2 (NimaGen, Nijmegen, The Netherlands) to construct an Illumina compatible sequence library. DNA sequencing was performed using 2x151 bp paired-end sequencing on a Illumina MiniSeq with a Mid-output sequence kit. Variant Calling and construction of the consensus sequence was performed using a custom designed easyseq pipeline (version 0.5.2) (30). To determine the lineage Pangolin (version 2.3.2) with pangoLEARN (version 2021-02-21) was used. Sequences were considered to belong to a cluster if they differ maximum 1 SNP from each other.

Centre 9

Extracted RNA was reverse trancribed using LunaScript RT (NEB), PCR amplicons were generated using IDT Midnight primers and Q5 High-Fidelity master mix (NEB). Transposase based fragmentation and barcode ligation was performed using the Ligation locost protocol (Oxford Nanopore Technologies).

Consensus fasta sequences were generated using the tools from the artic network (3). Read filtering was performed with guppyplex with the following paramters ` --skip-quality-check --min-length 900 --max-length 1600`. The output from guppyplex was used as input for the (nanopolish) artic minion pipeline, with `--normalise 200` as parameter. A custom scheme using primers of 1200bp was used (31).

Lineages were assigned using the command-line version (2.3.4) of pangolin (16). Clusters were identified with the command-line version of nextclade (0.14.1) with a threshold of less than 2 SNP difference. Input for both programs was the consensus fasta sequence generated by the artic minion pipeline.

Mean coverage was calculated with the command-line version (0.2.6) of mosdepth (32). The value under 'mean' for row 'total' was taken.

Centre 10

RNA was isolated using an easyMAG extractor following manufacturer's instructions for extraction of total nucleic acids from airways samples (BioMérieux, Marcy-l'Étoile, France). Detection of SARS-CoV-2 virus was performed using a validated qualitative RT-PCR detecting the SARS-CoV-2 virus E-gene based on a method published by Corman et al (28). Eluted RNA was reverse transcribed and PCR amplified according to the Artic Network v3 protocol using the ARTIC nCoV-2019 version 3 primer set with annealing temperature at 63 °C during PCR. The PCR products were sequenced on a GridION sequencer (Oxford Nanopore Technologies, Oxford, UK). The Medaka-pipeline by the ARTIC network (3) was used to generate consensus

sequences and call variant nucleotides relative to the reference sequence. Called variants were visualised in Geneious Prime (v2020.0.4) for validation and comparison. The consensus sequences were aligned using MAFFT and a phylogenetic tree using FastTree algorithm was generated to visualise the relatedness of the sequences in Geneious Prime. The criteria for samples being within an outbreak cluster was defined as sequences with < 3 SNPs differences.

Centre 11

RNA were extracted on a Biomek i7 automated workstation (Beckman Coulter) using their RNAdvanceViral kit (C63510) and protocol (and a Ct value from an in house Sarbeco-PCR provided). Further, we performed the Artic protocol v3 for PCR and library prep (1) using the ARTIC nCoV-2019 v3 primer panel from Integrated DNA technologies (Cat. No. 10006788), the Ligation sequencing kit (SQK-LSK109) and Native Barcoding Expansion 1-12 kit (EXP-NBD104) from Oxford Nanopore Technologies and ordered the 3. part reagents from New England Biolabs; Q5 Hot Start High-Fidelity 2X Master Mix (M0494L), LunaScript RT SuperMix Kit (E3010L), NEBNext® Ultra™ II End Repair/dA-Tailing Module (E7546L), NEBNext® Quick Ligation Module (E6056L) and Blunt/TA Ligase Master Mix (M0367L). The samples are loaded on a spot on Mk 1 R9 Version Flow Cell (Cat. No. FLO- MIN106D) and sequenced on a GridION device. For bioinformatic analysis, the fast5 files were basecalled and demultiplexed using guppy 4.3.4+ecb2805 on the GridION, with the flag to require barcodes on both ends turned on. We then used an in-house pipeline (33) which runs artic v1.2.1 (34) and then uses a QC script (35) to count number of aligned reads, base coverage and percentage of Ns. Any genomes with less than 90% of bases called with >20X reads are

then excluded, and lineage assignment is performed with pangolin (latest release) (16) and clade assignment with Nextclade CLI (latest release) (36).To define the clusters we compared the SNPs and deletions between the sequences belonging to the same lineages as reported by Nextclade. Sequences were deemed to belong to one cluster if they had maximally 0-1 SNP difference.

Centre 12

Nucleic acid extraction was performed using the Chemagic360™ platform and chemagic™ Viral DNA/RNA 300 Kit H96 extraction kit (PerkinElmer/Wallac, Turku, Finland). NGS library preparation was performed with QIAseq SARS-CoV-2 Primer Panel (QIAGEN, USA), the quality of the library was determined with QIAxcel DNA High Resolution Kit (QIAGEN) and Qubit™ dsDNA HS Assay Kit (Invitrogen™). Sequencing was performed with Illumina™ Miniseq platform using Miniseq Mid Output kit (300 cycles) (Illumina™, USA). Results were analyzed with Illumina BaseSpace application DRAGEN COVID Lineage and comparison was done with Nextclade software. The cluster assignment was based on the Nextclade and the DRAGEN COVID Lineage output.

Centre 13

Nucleic acid were extracted using the MagMAX Viral/Pathogen kit (Applied biosystems) from 200 ul of initial sample on a KingFisher Presto instrument (Thermo Fisher Scientific) integrated in the Nimbus Presto workstation (Hamilton). Nucleic acids were eluted in 50 ul and stored at -20°C before sequencing analysis. Then, 8.5 ul of eluates were used to prepare

the libraries using the Illumina COVIDSeq Test library preparation reagents (Illumina) according to the manufacturer's instructions. Libraries were sequenced on the Illumina NovaSeq 6000 SP flow cell, normally pooling 384 libraries per lane, using a 2x59-nt sequencing protocol. Paired reads were quality filtered and then analysed using an in-house processing pipeline developped by the Health 2030 Genome Center in Geneva (37).
Identification of clusters: complete genomes were automatically translated into proteins. Spike proteins were aligned using MAFFT and a phylogenetic Neighbour Joining tree was calculated. The clusters in the tree were identified by comparing signature substitutions/deletions in the alignment.

Centre 14

RNA was extracted using the MagDEA Dx SV kit on Maglead platform (PSS bio system net) according to manufacturer's instructions. A volume of 280ul lysis buffer was added to 220ul sample, and eluted in 50 µL. Sequencing libraries were prepared using the Illumina COVIDSeq Test, and sequenced on Novaseq 6000 producing at least 3.3 million paired end reads (150nt) per library.

Library quality was analyzed using FastQC (version 0.11.8, Babraham Bioinformatics). Reads were aligned to the genome using Bowtie2 (version 2.3.4.3) with the command options: -k 4 --no-discordant. reads with more than 6 variants in 100 bases were discarded (SNV, deletion or insertion each count as one variant). Variants were called using ivar variants (version 1.3.1). Consensus sequence was built based on the ivar variants table using the R Biostrings package according to these rules: Positions with less than 10 reads were called as N.

Variants with frequency higher than 0.7 were included in the consensus sequence. Variants with frequency between 0.3 and 0.7 and at least 50 reads were considered as "wobbles" using the IUPAC letters. Consensus sequences were submitted to Pangolin command-line tool (pangolin version 2.2.2 and pangoLEARN version 2021-02-12) and Nextclade (version 0.12.0) to determine the PANGO lineage and clade. Consensus sequences were aligned and a phylogenetic tree was built using ngphylogeny.fr – PhyML+SMS workflow, which is based on a maximum likelihood reference. Cluster identification was determined by samples having a shared ancestor on phylogenetic tree.

Centre 15

Nucleic acids were extracted using the MagNA Pure 96 system and the DNA and viral RNA small volume kit (Roche Diagnostics, Rotkreuz, Switzerland) or using the Abbott m2000 Realtime System and the Abbott sample preparation system reagent kit (Abbott, Baar, Switzerland). Amplicon sequencing followed the ARTIC nCOV-2019 protocol with a weighted v3 primer mix. Libraries were prepared with the Illumina DNA Prep kit (Illumina) on a Hamilton STAR robot. Up to 96 samples were pooled equimolarly and sequenced paired-end 150bp on an Illumina NextSeq 500 mid output flow cell.

Reads were demultiplexed with bcl2fastq v.2.17 (Illumina) and assembled using the COVGAP Pipeline (v10.6) (38) as previously described in (39,40). Briefly, a minimal depth of 50 was required for bases to be called. SNPs were called with a minimum allele frequency of 0.7. while ambiguous bases with lower allele frequency were masked for further analysis.

Clusters were identified by calculating a maximum likelihood tree using RAxML with a maximum difference of 1 SNP between sequences.

References

1. Quick J. nCoV-2019 sequencing protocol v3 (LoCost) V.3. https://www.protocols.io/view/ncov-2019-sequencing-protocol-v3-locost-bh42j8ye. 2020.
2. SmaltAlign. https://github.com/medvir/SmaltAlign.
3. Loman N, Rambaut A. nCoV-2019 novel coronavirus bioinformatics protocol. https://artic.network/ncov-2019/ncov2019-bioinformatics-sop.html.
4. Grädel C, Miani MAT, Barbani MT, Leib SL, Suter-Riniker F, Ramette A. Rapid and cost-efficient enterovirus genotyping from clinical samples using flongle flow cells. Genes. 2019;10(9).
5. Neuenschwander SM, Miani MAT, Amlang H, Perroulaz C, Bittel P, Casanova C, et al. A sample-to-report solution for taxonomic identification of cultured bacteria in the clinical setting based on nanopore sequencing. Journal of Clinical Microbiology. 2020;58(6).
6. Medaka. https://github.com/nanoporetech/medaka.
7. Li H. A statistical framework for SNP calling, mutation discovery, association mapping and population genetical parameter estimation from sequencing data. Bioinformatics. 2011;27(21).
8. GENCOV. https://github.com/metagenlab/GENCOV.
9. CoVpipe. https://gitlab.com/RKIBioinformaticsPipelines/ncov_minipipe.
10. Chen S, Zhou Y, Chen Y, Gu J. Fastp: An ultra-fast all-in-one FASTQ preprocessor. In: Bioinformatics. 2018.
11. Li H. Aligning sequence reads, clone sequences and assembly contigs with BWA-MEM. arXiv preprint arXiv. 2013;
12. Okonechnikov K, Conesa A, García-Alcalde F. Qualimap 2: Advanced multi-sample quality control for high-throughput sequencing data. Bioinformatics. 2016;32(2).
13. fgbio. https://github.com/fulcrumgenomics/fgbio.
14. Garrison E, Marth G. Haplotype-based variant detection from short-read sequencing -- Free bayes -- Variant Calling -- Longranger. arXiv preprint arXiv:12073907. 2012;
15. Danecek P, Bonfield JK, Liddle J, Marshall J, Ohan V, Pollard MO, et al. Twelve years of SAMtools and BCFtools. GigaScience. 2021;10(2).
16. Rambaut A, Holmes EC, O'Toole Á, Hill V, McCrone JT, Ruis C, et al. A dynamic nomenclature proposal for SARS-CoV-2 lineages to assist genomic epidemiology. Nature Microbiology. 2020;5(11).
17. ARTIC nanopore protocol for nCoV2019 novel coronavirus. https://github.com/artic-network/artic-ncov2019.
18. Jalili V, Afgan E, Gu Q, Clements D, Blankenberg D, Goecks J, et al. The Galaxy platform for accessible, reproducible and collaborative biomedical analyses: 2020 update. Nucleic Acids Research. 2021;48(W1).
19. Maier W, Bray S, van den Beek M, Bouvier D, Coraor N, Miladi M, et al. Freely accessible ready to use global infrastructure for SARS-CoV-2 monitoring. bioRxiv. 2021;
20. Li H, Durbin R. Fast and accurate short read alignment with Burrows-Wheeler transform. Bioinformatics. 2009;25(14).
21. iVar. https://andersen-lab.github.io/ivar/html/manualpage.html.

22. Wilm A, Aw PPK, Bertrand D, Yeo GHT, Ong SH, Wong CH, et al. LoFreq: A sequence-quality aware, ultra-sensitive variant caller for uncovering cell-population heterogeneity from high-throughput sequencing datasets. Nucleic Acids Research. 2012;40(22).
23. Cingolani P, Platts A, Wang LL, Coon M, Nguyen T, Wang L, et al. A program for annotating and predicting the effects of single nucleotide polymorphisms, SnpEff: SNPs in the genome of Drosophila melanogaster strain w1118; iso-2; iso-3. Fly. 2012;6(2).
24. Li H, Handsaker B, Wysoker A, Fennell T, Ruan J, Homer N, et al. The Sequence Alignment/Map format and SAMtools. Bioinformatics. 2009;25(16).
25. GitHub: SARS-CoV-2 Analyses. https://github.com/jonas-fuchs/SARS-CoV-2-analyses.
26. Oude Munnink BB, Nieuwenhuijse DF, Stein M, O'Toole Á, Haverkate M, Mollers M, et al. Rapid SARS-CoV-2 whole-genome sequencing and analysis for informed public health decision-making in the Netherlands. Nature Medicine. 2020;26(9).
27. MACOVID. https://github.com/MUMC-MEDMIC/MACOVID.
28. Corman VM, Landt O, Kaiser M, Molenkamp R, Meijer A, Chu DKW, et al. Detection of 2019 novel coronavirus (2019-nCoV) by real-time RT-PCR. Eurosurveillance. 2020;25(3).
29. Coolen JPM, Wolters F, Tostmann A, van Groningen LFJ, Bleeker-Rovers CP, Tan ECTH, et al. SARS-CoV-2 whole-genome sequencing using reverse complement PCR: For easy, fast and accurate outbreak and variant analysis. Journal of Clinical Virology. 2021;144.
30. Easyseq. https://github.com/JordyCoolen/easyseq_covid19.
31. Freed NE, Vlková M, Faisal MB, Silander OK. Rapid and inexpensive whole-genome sequencing of SARS-CoV-2 using 1200 bp tiled amplicons and Oxford Nanopore Rapid Barcoding. Biology Methods and Protocols. 2021;5(1).
32. Pedersen BS, Quinlan AR. Mosdepth: Quick coverage calculation for genomes and exomes. Bioinformatics. 2018;34(5).
33. SusCovONT. https://github.com/marithetland/susCovONT.
34. The ARTIC field bioinformatics pipeline. https://github.com/artic-network/fieldbioinformatics.
35. Connor lab: QC script. https://github.com/connor-lab/ncov2019-artic-nf/blob/master/bin/qc.py.
36. Aksamentov I, Neher R. Nextclade. https://github.com/nextstrain/nextclade. 2020.
37. Health 2030 Genome Center SARS-CoV2 pipeline. https://github.com/health2030genomecenter/SARS-CoV-2_pipeline.
38. Mari A. COVGAP. https://github.com/appliedmicrobiologyresearch/covgap.
39. Stange M, Marii A, Roloff T, Seth-Smith HMB, Schweitzer M, Brunner M, et al. SARS-CoV-2 outbreak in a tri-national urban area is dominated by a B.1 lineage variant linked to a mass gathering event. PLoS Pathogens. 2021;17(3).
40. Mari A, Roloff T, Stange M, Søgaard KK, Asllanaj E, Tauriello G, et al. Global Genomic Analysis of SARS-CoV-2 RNA Dependent RNA Polymerase Evolution and Antiviral Drug Resistance. Microorganisms. 2021 May 19;9(5).
