# Supplementary material for: External Quality Assessment of SARS-CoV-2 Sequencing: an ESGMD-SSM Pilot Trial across 15 European Laboratories
Source: J Clin Microbiol. 2022 Jan 19;60(1):e01698-21. doi: 10.1128/JCM.01698-21 (PMC8769736; doi:10.1128/JCM.01698-21)
Supplement: Supplemental file 3 — Text S3. Download JCM.01698-21-s0003.pdf, PDF file, 146 KB [file jcm.01698-21-s0003.pdf]

Supplemental Tables

Sample	Ct
NGS1	22
NGS2	21.5
NGS3	20.8
NGS4	19.4
NGS5	19.9
NGS6	21.1
NGS7	27.1
NGS8	28
NGS9	28.4
NGS10	28.1

Suppl. table S1: Samples and viral load as measured by qPCR provided to the participating laboratories.

Centers	NGS1	NGS2	NGS3	NGS4	NGS5	NGS6	NGS7	NGS8	NGS9	NGS10	Mean	SD
1	3080	3628	3051	3546	3200	4183	3780	3268	4468	4347	3655	527
2	406	412	409	413	417	408	393	349	391	383	398	21
3	7558	7493	7915	7099	7132	8061	7462	7755	7985	7907	7637	344
4	3011	3131	2898	3659	3669	3320	2618	1583	2069	862	2682	918
5	2999	2766	3678	3696	2597	5062	3600	2556	3346	2124	3242	835
6	2996	1752	2854	2930	2112	2440	1917	2996	3113	2819	2593	500
7	862	191	139	171	131	240	525	39	703	246	325	275
8	896	807	1414	982	1222	1047	1346	260	579	424	898	386
9	392	396	301	400	412	375	361	9	334	148	313	132
10	1196	1209	1210	1223	1210	1215	1186	796	1077	1082	1140	132
11	960	963	690	932	908	1028	1038	448	888	1017	887	184
12	3523	2990	2893	2759	2890	3014	2556	3078	3366	3510	3058	320
13	6044	5012	5093	6052	6316	6079	6119	5530	5844	6144	5823	457
14	39294	36491	29619	41782	37390	36710	42894	36949	34715	35877	37172	3712
15	2355	2256	2529	2680	2864	2685	1175	1322	1660	1364	2089	644

Suppl. table S2: Mean read depth for each sample and centre.

Centre	NGS1	NGS2	NGS3	NGS4	NGS5	NGS6	NGS7	NGS8	NGS9	NGS10
1	0.28	0.28	0.28	0.28	0.28	0.27	0.27	0.28	0.27	0.52
2	0.00	0.00	0.84	1.58	0.00	0.84	0.00	2.61	1.79	1.74
3	0.02	0.02	0.02	0.01	0.03	0.02	0.04	0.12	0.05	0.07
4	0.26	0.22	0.22	0.22	0.22	0.22	0.22	0.45	0.29	1.12
5	0.11	0.11	0.41	0.10	0.10	0.10	0.10	1.30	0.12	0.69
6	0.80	0.65	3.02	0.00	0.00	0.00	0.65	0.64	1.39	0.65
7	2.63	1.64	9.31	1.19	1.44	1.06	6.21	43.18	3.39	7.95
8	0.62	0.62	0.62	0.62	0.62	0.62	0.65	1.15	0.74	1.19
9	0.63	0.63	3.26	3.40	0.63	3.26	3.98	99.91	7.49	0.64
10	0.40	0.41	0.40	0.40	0.40	0.40	0.40	2.24	0.40	1.34
11	1.40	0.41	2.24	1.99	0.40	1.26	0.42	5.80	3.35	1.34
12	0.27	0.13	0.14	0.14	0.13	0.13	0.68	1.37	0.13	0.75
13	0.25	0.25	0.25	0.25	0.25	0.25	0.25	0.69	0.25	1.03
14	0.26	0.27	0.27	0.24	0.26	0.56	0.26	0.96	0.27	1.08
15	0.30	0.30	1.04	0.31	0.30	1.04	0.30	4.61	1.26	1.30

Suppl. table S3: Percentage of missing data (Ns) in consensus genomes.

Centre	NGS1	NGS2	NGS3	NGS4	NGS5	NGS6	NGS7	NGS8	NGS9	NGS10	mean
1	0.83	0.98	1.00	0.92	1.00	1.00	0.78	0.83	0.81	0.62	0.88
2	0.93	0.95	0.94	0.92	1.00	0.94	0.78	0.71	0.77	0.54	0.85
3	0.87	0.95	0.94	0.92	1.00	0.94	0.78	0.86	0.81	0.63	0.87
4	0.93	1.00	1.00	1.00	1.00	1.00	0.97	0.86	1.00	0.95	0.97
5	0.93	1.00	0.89	1.00	1.00	0.94	0.97	0.71	1.00	0.88	0.93
6	0.93	0.95	1.00	0.92	1.00	1.00	0.81	0.86	0.84	0.63	0.89
7	0.87	1.00	0.56	0.92	1.00	0.83	0.69	0.24	0.81	0.63	0.75
8	0.93	1.00	0.89	0.88	1.00	0.89	0.69	0.76	0.71	0.63	0.84
9	0.93	1.00	0.94	0.92	1.00	0.94	0.75	0.00	0.74	0.63	0.79
10	0.93	0.95	1.00	0.92	1.00	1.00	0.78	0.81	0.81	0.54	0.87
11	0.87	1.00	0.94	0.92	1.00	0.94	0.63	0.81	0.81	0.61	0.85
12	0.93	1.00	1.00	0.92	1.00	1.00	0.75	0.76	0.81	0.56	0.87
13	0.93	1.00	1.00	1.00	1.00	1.00	0.97	0.81	1.00	0.90	0.96
14	0.87	1.00	1.00	1.00	1.00	0.94	0.97	0.71	1.00	0.90	0.94
15	0.93	1.00	0.94	1.00	1.00	0.94	0.97	0.57	1.00	0.78	0.91
mean	0.91	0.99	0.94	0.94	1.00	0.96	0.81	0.69	0.85	0.68	

Suppl. table S4: Variant calling score for each sample and centre and mean score per centre.

centre	NGS1	NGS2	NGS3	NGS4	NGS5	NGS6	NGS7	NGS8	NGS9	NGS10
1	13	2	0	20	1	0	18	0	0	23	1	0	20	0	0	18	0	0	28	3	0	20	1	0	28	3	0	31	7	1
2	15	0	0	20	0	1	17	0	1	23	1	0	20	0	0	17	0	1	28	3	0	18	0	3	27	3	1	29	7	3
3	13	0	2	20	0	1	17	0	1	23	1	0	20	0	0	17	0	1	28	3	0	21	0	0	28	3	0	32	6	1
4	15	0	0	21	0	0	18	0	0	24	0	0	20	0	0	18	0	0	31	0	0	21	0	0	31	0	0	39	0	0
5	14	0	1	21	0	0	16	0	2	24	0	0	20	0	0	17	0	1	31	0	0	19	1	1	31	0	0	36	0	3
6	15	0	0	20	0	1	18	0	0	23	1	0	20	0	0	18	0	0	28	2	1	21	0	0	28	2	1	32	6	1
7	14	0	1	21	0	0	11	1	6	22	0	2	20	0	0	16	1	1	25	3	3	8	0	13	27	2	2	31	5	3
8	15	0	0	21	0	0	17	1	0	22	1	1	20	0	0	17	1	0	26	4	1	20	1	0	26	4	1	32	6	1
9	15	0	0	21	0	0	17	0	1	23	1	0	20	0	0	17	0	1	27	3	1	0	0	21	26	3	2	32	6	1
10	15	0	0	20	0	1	18	0	0	23	1	0	20	0	0	18	0	0	28	3	0	20	0	1	28	3	0	29	7	3
11	14	0	1	21	0	0	17	0	1	23	1	0	20	0	0	17	0	1	23	3	5	18	0	3	28	3	0	31	6	2
12	15	0	0	21	0	0	18	0	0	23	1	0	20	0	0	18	0	0	27	3	1	20	1	0	28	3	0	30	7	2
13	15	0	0	21	0	0	18	0	0	24	0	0	20	0	0	18	0	0	31	0	0	20	0	1	31	0	0	36	0	2
14	14	1	0	21	0	0	18	0	0	24	0	0	20	0	0	17	0	1	31	0	0	18	2	1	31	0	0	39	0	0
15	15	0	0	21	0	0	17	0	1	24	0	0	20	0	0	17	0	1	31	0	0	17	3	1	31	0	0	33	1	5

Suppl. Table S5: Count of (correct | wrong | missing) SNP calls for each sample and centre.
